# Salivary cortisol reactivity to stress in pregnancy: A scoping review

**DOI:** 10.1016/j.psyneuen.2026.107895

**Published:** 2026-05-16

**Authors:** Georgia F. Celestin, Maria Restrepo, Madeline Daugherty, Lauren A. Costello, Özlü Aran, Philip Espinola-Coombs, Brie M. Reid

**Affiliations:** a Department of Psychology, Northeastern University, 125 Nightingale Hall, 360 Huntington Ave, Boston, MA 02115, USA; b Institute of Cognitive and Brain Health, Northeastern University, 360 Huntington Ave, Boston, MA 02115, USA; c Department of Public Health and Health Sciences, Northeastern University, 360 Huntington Ave, Boston, MA 02115, USA; d Research & Instruction, Northeastern University Library, 360 Huntington Ave, Boston, MA 02115, USA

**Keywords:** Cortisol reactivity, Stress, Pregnancy, Saliva, Review

## Abstract

**Objective(s):**

Cortisol reactivity to acute experimental stressors indexes hypothalamic-pituitary-adrenal (HPA) axis responsivity under challenge, a dimension of stress physiology that is particularly consequential during pregnancy, when neuroendocrine adaptations alter how the stress system functions. No scoping review has characterized this literature. The present study mapped evidence on salivary cortisol reactivity to acute experimental stressors during pregnancy, with attention to experimental paradigms, mental health, psychological stress, gestational timing, and demographic characteristics.

**Method:**

A systematic search yielded 1234 records, of which 33 empirical studies representing 2418 pregnant participants met inclusion criteria. Data were extracted and charted using a standardized framework. Cortisol data were digitized from published figures where necessary for descriptive visualization.

**Results:**

Fewer than half of studies (39.4%) found significant cortisol responses to the administered stressor. Social stressors elicited significant responses more consistently than non-social stressors (47.4% vs. 33.3%). Associations between cortisol reactivity and depressive symptomatology were found in less than half of tests, with all significant associations in the positive direction. Substantial heterogeneity in sampling timing, pre-testing protocols, and operationalization of reactivity was observed across studies. There was limited ability to compare cortisol reactivity across racial and ethnic identity, socioeconomic variables, or gestational age.

**Conclusions:**

This scoping review of salivary cortisol reactivity during pregnancy reveals a growing literature marked by substantial methodological heterogeneity that limits cross-study synthesis. Social-evaluative stressors are recommended to improve HPA axis activation in pregnancy. Future research should prioritize diverse populations, trimester-specific reporting, and standardized cortisol reporting (means, standard deviations, sampling times) at each time point relative to stressor onset.

## Graphical Abstract

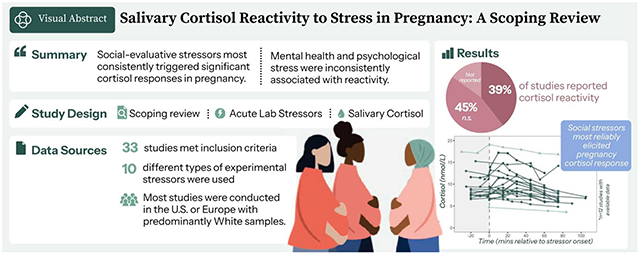

## 1. Introduction

Cortisol reactivity to acute experimental stressors indexes hypothalamic–pituitary–adrenal (HPA) axis responsivity under challenge—a dimension of stress physiology that is especially consequential during pregnancy, when neuroendocrine adaptations fundamentally alter how the stress system functions. Despite a growing body of empirical work over the last few decades, studies vary substantially across experimental paradigms, populations, and reactivity operationalizations. Given this heterogeneity, a scoping review is particularly well-suited to map the existing evidence, identify gaps, and determine where future synthesis efforts should be directed. To date, no scoping review has characterized the literature on salivary cortisol reactivity to acute stress during pregnancy. The present review addresses this gap and examines the current literature’s experimental paradigms, gestational timing, and population characteristics to identify what is known, where inconsistencies lie, and where future research is most needed.

Pregnancy involves profound neuroendocrine adaptations that distinguish cortisol regulation during gestation from those observed in non-gravid adults, in whom cortisol release following stressor exposure is regulated by negative feedback mechanisms that restore the HPA axis to homeostatic levels (Lupien et al., 2009). Circulating cortisol concentrations increase markedly over the course of pregnancy, reaching levels seven times higher than those observed outside of pregnancy (Howland et al., 2017). Cortisol responses to acute stressors are often attenuated during pregnancy, a pattern thought to reflect normative adaptations that may protect both the pregnant individual and the developing fetus from sustained glucocorticoid exposure (de Weerth and Buitelaar, 2005; Glynn et al., 2001, 2004). These gestational changes in cortisol regulation are driven in part by the emergence of the placenta as a central endocrine organ, which facilitates the exchange between the maternal and fetal units while producing its own hormones that regulate maternal physiology (Howland et al., 2017). One of these hormones, placental corticotropin-releasing hormone (CRH), is the main regulator of stress-related hormone production during pregnancy (Goland et al., 1994; Sandman, 2018). Critically, placental CRH production is stimulated rather than suppressed by cortisol, resulting in a positive feedback loop that contributes to escalating cortisol concentrations across pregnancy.

Cortisol reactivity to acute stress can exert widespread influence on health outcomes relevant to the gestating individual and fetus (Coussons-Read et al., 2007; Hantsoo et al., 2019; Lupien et al., 2009; Reid, 2024). In non-pregnant populations and animal models, cortisol reactivity is associated with health behaviors, depressive symptoms, cognitive performance, and inflammation (Chang et al., 2022; Kunz-Ebrecht et al., 2003; Lopresti, 2020; Morris et al., 2012; Souza-Talarico et al., 2011). In pregnancy, dampened stress responses may be adaptive and potentially protect the maternal-fetal unit from adverse birth outcomes such as preterm birth (Glynn et al., 2008). Conversely, changes to stress reactivity – whether hyper- or hypo-reactive—may reflect physiological adaptations to chronic stress exposure, with downstream consequences for health (Young et al., 2021). This literature supports cortisol reactivity as a meaningful index of stress system function with relevance to multiple health domains.

In 2005, de Weerth and Buitelaar published the first review of physiological stress reactivity in human pregnancy, spanning heart rate, blood pressure, and cortisol—though only three of fifteen included papers measured cortisol, and just one used salivary sampling (de Weerth and Buitelaar, 2005). A subsequent narrative review by Christian (Christian, 2012) focused specifically on the health implications of stress reactivity in pregnancy, including five studies that used salivary cortisol. Across both reviews, the authors highlight how stress reactivity provides unique insight into prenatal programming and fetal effects. However, this literature has expanded substantially in the intervening years, alongside advances in experimental paradigms, analytic approaches, and the inclusion of more diverse populations. While diurnal cortisol measures in pregnancy have been linked to fetal development and offspring psychopathology risk (Hantsoo et al., 2019; Monk et al., 2019), they index cortisol secretion under relatively neutral conditions that are shaped by circadian processes (Rinne et al., 2023). In contrast, cortisol reactivity (also called ‘phasic’ cortisol) captures dynamic HPA axis responses to acute challenge and may be particularly informative in pregnancy given gestational alterations in baseline cortisol and stress responsivity.

Characterizing stressor type is important for interpreting cortisol reactivity findings. In the 2005 review (de Weerth and Buitelaar, 2005), stressors were split into two groups: physical (pain) and psychological categories, but the authors were unable to draw a conclusion about differential effects by stressor type, particularly because of substantial methodological variability across studies. This is a meaningful gap, as meta-analytic work in non-pregnant populations shows that stressors involving social-evaluative threat and uncontrollability (situations in which the self can be negatively judged by others) provoke more consistent cortisol responses among participants as compared to tasks lacking those characteristics (Biondi and Picardi, 1999; Dickerson and Kemeny, 2004; Kudielka et al., 2009). Stressor type was not examined in the 2012 review (Christian, 2012). As such, characterization of cortisol reactivity to both social and non-social stressors in pregnant populations remains lacking.

Beyond stressor type, methodological heterogeneity across studies poses a challenge for synthesizing this literature. Since the inaugural study measuring salivary cortisol reactivity in pregnancy in 2002 (Kammerer et al., 2002), salivary sampling has become the dominant approach. Since 2002, experimental assessment of cortisol reactivity during pregnancy has relied primarily on salivary cortisol sampling because it provides a non-invasive measure of free, biologically active cortisol and is well suited to repeated sampling designs (Kirschbaum, 1992; Kudielka et al., 2009). However, studies vary considerably in sampling timing, number of samples, and how reactivity is operationalized. These differences matter because the HPA axis exhibits temporal constraints on stress responsivity: cortisol typically peaks ~20–30 min following stressor onset, with limited capacity for repeated activation within short time windows (Gunnar et al., 2021; Lopez-Duran et al., 2014). Variability in whether and how studies account for these constraints contributes to inconsistency in findings across the literature.

Characterizing participants using psychosocial and other demographic variables to identify patterns has also increased in recent decades in pregnancy research after calls to report on demographics in psychology research more broadly (O’Bryant et al., 2004). Although de Weerth and Buitelaar (2005) did not discuss participant demographics or other external factors that may modulate reactivity, the authors suggested using psychological and personality variables during testing sessions to assess stress perception. In the 2012 review, Christian identified the lack of work assessing physiological reactivity in pregnancy by psychological variables, gestational age, and race, as critical gaps in knowledge. Exposure to chronic and cumulative stressors prior to and during pregnancy may shape HPA axis calibration, raising the possibility that normative attenuation of cortisol reactivity during pregnancy may not be uniform across populations with experiences of structural disadvantage, early adversity, or clinical risk. A 2021 integrative review focused on early life adversity and multiple HPA parameters in pregnancy reported that exposure to early life adversity was associated with blunted cortisol reactivity to acute stress during pregnancy, even after accounting for current stress and psychosocial symptoms (Epstein et al., 2021). Additionally, a recent scoping review characterized associations between maternal stress and diurnal measures of cortisol across pregnancy, but it did not include any empirical studies assessing cortisol reactivity (Rinne et al., 2023). Taken together, these gaps underscore the need for a comprehensive synthesis of cortisol reactivity to acute stress in pregnancy that attends to psychosocial context, demographic variability, and gestational timing.

The present scoping review maps the existing literature on salivary cortisol reactivity to acute experimental stressors during pregnancy. We characterize experimental paradigms, cortisol reactivity operationalizations, and patterns of responsivity across trimesters and populations, to identify methodological gaps and inform future research on stress physiology in human pregnancy. By establishing the scope and structure of this literature, this review is intended as a foundation for future systematic reviews and meta-analyses that can more precisely quantify effects and test hypotheses about when, for whom, and under what conditions cortisol reactivity is observed during pregnancy.

## 2. Methods

Scoping reviews are an evidence synthesis methodology which seeks to uncover gaps and patterns within the existing literature on a specific topic (Munn et al., 2018). This scoping review was developed with reference to the JBI Scoping Review Methodology Group’s guidance on conducting scoping reviews and reported following the Preferred Reporting Items for Systematic Reviews and Meta-Analyses- Extension for Scoping Reviews (PRISMA-ScR) reporting guideline (Peters et al., 2024; Tricco et al., 2018). The protocol was registered on OSF Registries (Celestin et al., 2025).

### 2.1. Eligibility criteria

To be included in the present review, papers must report on salivary cortisol reactivity in pregnancy. Papers were included if they were: published between 1990 and 2025, written in English, measured cortisol reactivity using saliva, used an induced/experimental stressor, had at least one saliva measurement pre-stressor and one saliva measurement post-stressor, used pregnant participants and reported their gestational age, and were a primary research paper. Papers were excluded if there was no administered stressor, if they were a review, editorial, or other text without a full-length article, if cortisol was measured using something other than saliva (e.g., plasma, hair), if they were not in pregnancy, or if there was less than one measure of cortisol pre- and post-stressor. Our inclusion criterion for publication year began in 1990, as the first publication using salivary cortisol measurement (non-reactivity) in human pregnancy was published in this year (Allolio et al., 1990).

### 2.2. Search strategy

The following sources were searched on March 5, 2025: PubMed, Embase via Embase.com, CINAHL Ultimate via EBSCO, PsycINFO via EBSCO, Web of Science Core Collection, Scopus, and Cochrane CENTRAL. The full database search strings can be found in Appendix A.

### 2.3. Study selection & data extraction

Removal of duplicate results, study selection, and data extraction were all conducted using (Covidence Veritas Health Innovation, 2025).

Reviewers conducted a pilot screening of six titles and abstracts to ensure clarity of the eligibility criteria. Two independent reviewers participated in the title and abstract screening, as well as the full text screening. Discrepancies in selection were adjudicated by consensus.

Dual independent data extraction was performed. Discrepancies were adjudicated by a third reviewer. The following data points were extracted: study ID, title, year of publication, corresponding author contact details, country in which the study was conducted, study design, participant races/ethnicities, number of participants by race/ethnicity (if reported), presence of subgroups, whether or not household income data was reported, participants’ pregnancy history, gestational ages represented, gestational age (mean, standard deviation, range), maternal age (mean, standard deviation, range), mental health measures used, total number of participants, stressor used, total number of saliva collections, whether means were reported on each saliva sample, saliva collection method used, timing of sample collection, whether participants were instructed to eat or drink, cortisol data transformation (e.g. log base 10, natural log), cortisol data unit, how ‘reactivity’ was operationalized, and variables associated with cortisol.

### 2.4. Data analysis

To provide descriptive context for the reviewed literature, cortisol data were extracted and visualized across studies for illustrative purposes only— not to support quantitative synthesis or meta-analytic conclusions. Extracted data were exported from Covidence as a CSV file and subsequently summarized using R. Significant cortisol responding was determined based on author reporting within each individual study. When numerical cortisol data were not reported in text or tables (n = 29), values were extracted from published figures where possible (n = 13) using PlotDigitizer (PlotDigitizer, 2025), an AI-assisted graph digitization software with established validity, reliability, and higher interrater agreement than manual extraction (Aydin and Yassikaya, 2022; Jelicic Kadic et al., 2016). Figures were imported into the software, axes were calibrated based on the scales provided in each study, and data points or summary estimates were digitized and visually inspected. Extracted values were cross-checked against the original graphical representations to ensure accuracy. Where necessary, sampling times were adjusted so that stressor onset was presented as zero on the x-axis. All digitized cortisol values were subsequently converted into a standardized unit of nanomoles per liter (nmol/L) using established conversion factors to facilitate comparability across studies, and extraction times were rounded to the nearest whole number and verified against reported collection times in each study's methods. Digitized values were used solely for visualization and descriptive comparison across studies and were not included in any statistical analyses. Variance estimates were not incorporated into visualizations because they were sparsely and inconsistently reported across studies: among those that did report them, the type of estimate varied (e.g., standard deviation, standard error, confidence intervals), and sample sizes underlying figure-level means could not be reliably established. Values are therefore presented as unweighted study-level means without error bars or any form of statistical aggregation. Importantly, the extracted data were not aggregated or modified in any way beyond unit conversion but were instead plotted to allow direct visual comparison across studies within individual figures. Statistical analysis or interpretation of the visualized data is beyond the scope of this scoping review.

## 3. Results

**Fig. 1.**
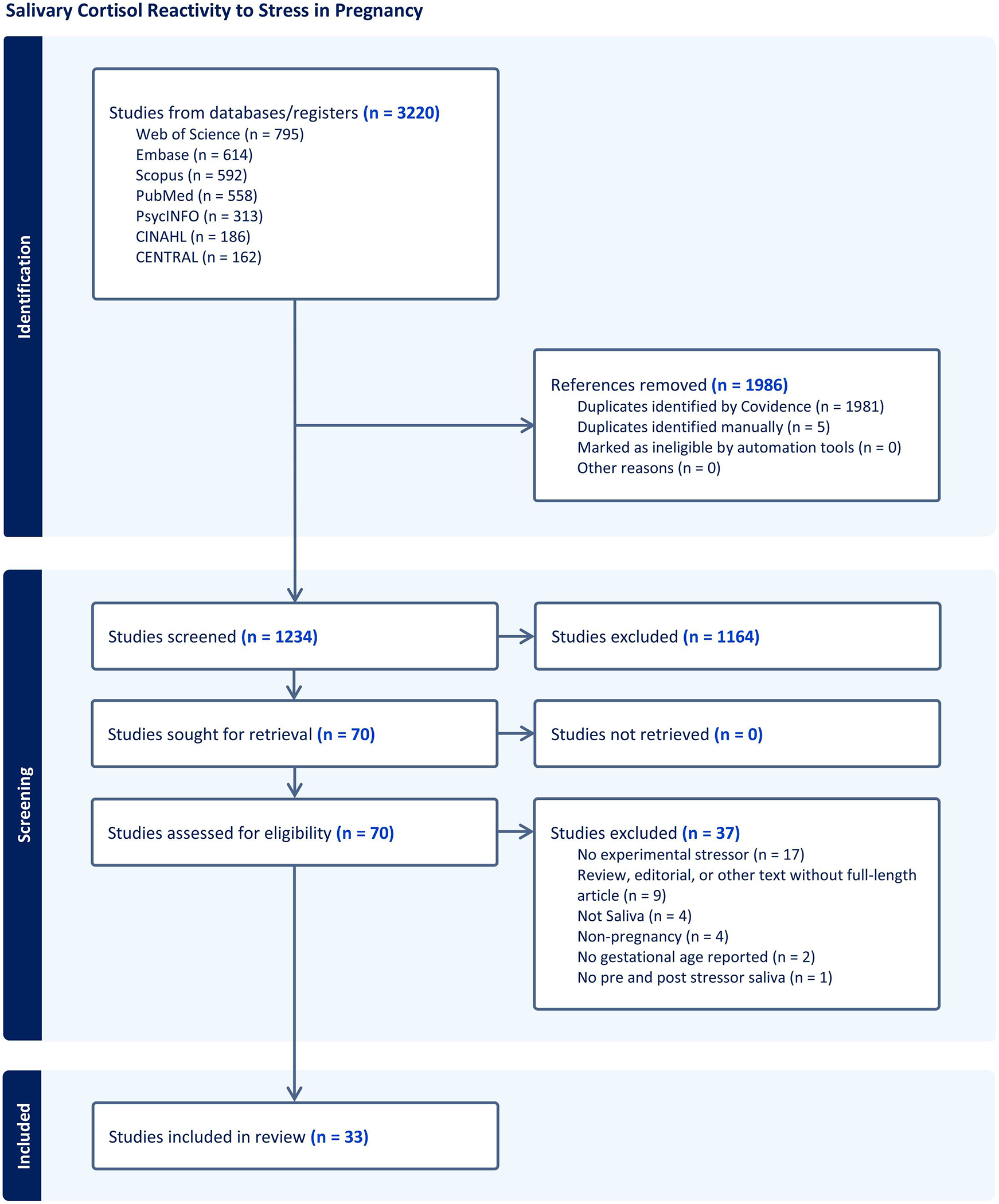
PRISMA flow diagram.

After import and deduplication of results, 1234 records were screened at the title and abstract level. Of those, 70 studies were assessed at the full text level. In total, 33 studies met the eligibility criteria and were included in this review. Full data can be seen in the PRISMA flow chart (Fig. 1).

### 3.1. Description of included studies

Of the 33 studies (summarized in Table 1), most were conducted in the United States (n = 17) followed by Switzerland (n = 7), the United Kingdom (n = 3) and the Netherlands (n = 2). One study each was conducted in Türkiye, Israel, China, and Austria. Nearly half of studies were cross-sectional (n = 16) while the remaining were longitudinal or cohort studies (n = 11), randomized controlled trials (n = 4), a crossover (n = 1), or intervention to treatment study (n = 1). The total sample sizes ranged from 10 participants (Kammerer et al., 2002) to 255 participants (Levendosky et al., 2024) with a mean sample size of 75.33 (SD = 58.32). Most studies (n = 20) had subgroups within their samples (i.e., splitting participants based on various demographic or diagnostic factors for cross comparison).

### 3.2. Participants

**Fig. 2.**
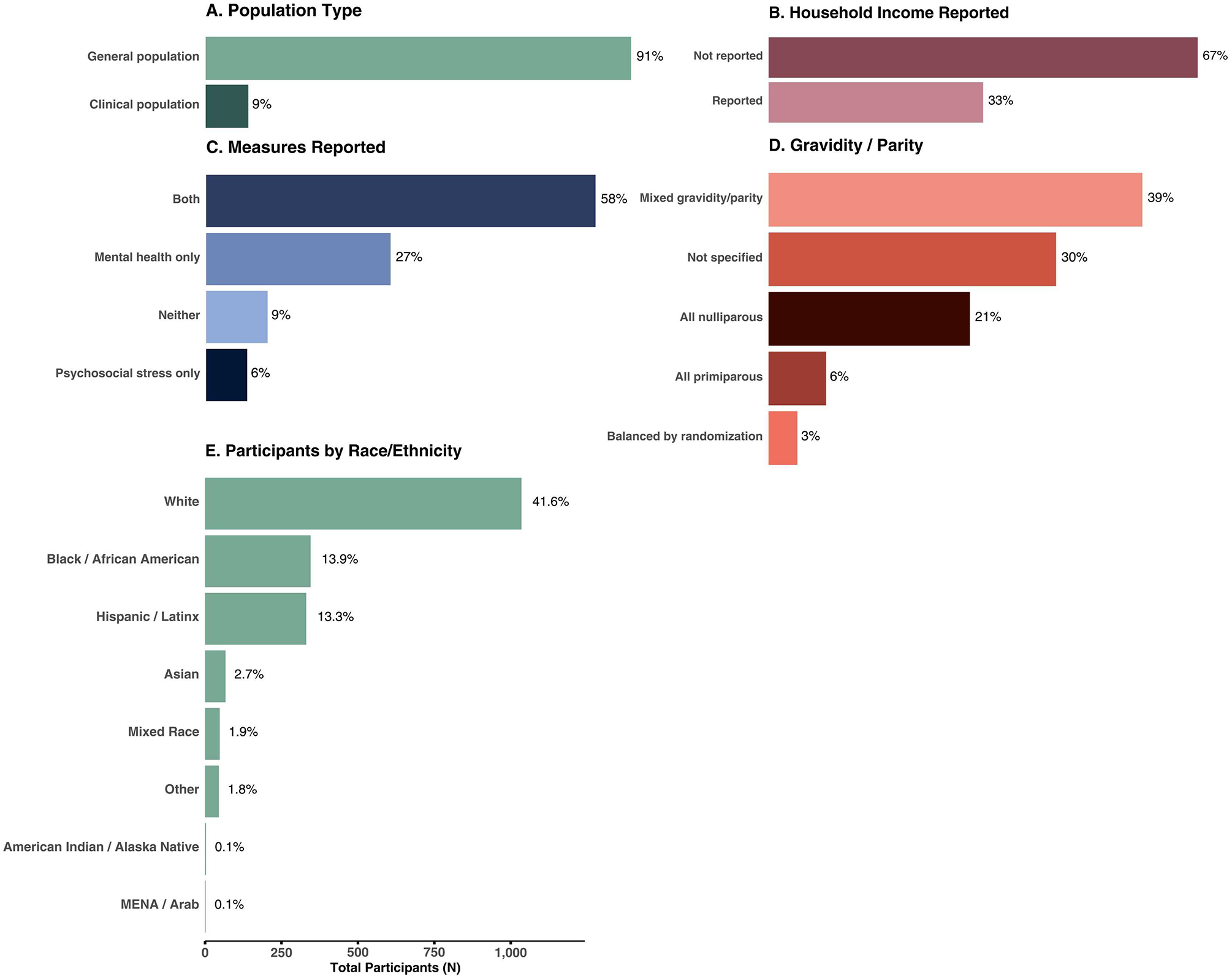
Study level characteristics of included studies. Panels show that most of the 33 studies: used non-clinical populations (A), did not report household income (B), reported both psychological stress and mental health measures (C), and used participants with mixed gravidity and/or parity (D). Panel (E) represents 21 studies and shows that the majority of included participants identified as White; bars show total N, labels show pooled percentage across studies. MENA – Middle Eastern/North African.

A total of 2418 pregnant participants are represented across the 33 included studies. Fig. 2 summarizes study-level characteristics of the included studies. Panel A shows that three studies used clinical populations, defined here as studies requiring participants to carry a specific diagnosis or seeking non-routine medical care for inclusion. Specifically, one study recruited participants referred by their obstetrician for antenatal fetal surveillance (Turan et al., 2024), another enrolled participants at high risk for depression due to a prior depression or anxiety diagnosis (Laurent et al., 2018), and a third required an anxiety diagnosis for inclusion (Riddle et al., 2023). Panel B shows that only one-third of studies reported household income data. Panel C illustrates that nearly all studies reported at least one mental health or psychological stress measure, with the majority (n = 19) reporting both. Panel D shows that most studies had mixed gravidity and parity across their sample (n = 13) or did not specify these characteristics (n = 10). Two studies used all primiparous participants (Braithwaite et al., 2015; Braithwaite et al., 2016) and seven used all nulliparous participants, including one for which primiparity was an inclusion criterion (Murphy et al., 2015). The remaining study used randomization to ensure a balance between primiparous and multiparous participants (Lustermans et al., 2024). Panel E shows the distribution of race and ethnicity across the 21 studies that reported these data. The remaining 12 studies (36.4%) did not report race and ethnicity data, including all non-US and non-UK studies with the exception of one Netherlands-based study that reported an exclusively White sample. Of the 31 studies (94%) that reported maternal age, the pooled mean was 28.68 years (SD = 5.25). Most studies included participants across more than one trimester (n = 18, 54.5%), while ten studies (30.5%) were restricted to the third trimester and five (15.2%) to the second trimester.

### 3.3. Measurement of salivary cortisol reactivity

Studies sampled salivary cortisol between two and nine times across their relative procedures (mean number of samples = 5.1). The majority (78.8%) of included studies used immunoassays for cortisol quantification (radioimmunoassay, n = 8; ELISA, n = 4; luminescence or chemiluminescence immunoassay, n = 5; enzyme immunoassay, n = 8; Time-resolved, n = 1). The remaining studies used multiplex assays (n = 1), highly sensitive liquid chromatography–tandem mass spectrometry (n = 3) or did not specify/report (n = 3). Out of the 21 studies (63.6%) that reported coefficients of variation for assays, all were within accepted ranges (10% or below).

**Fig. 3.**
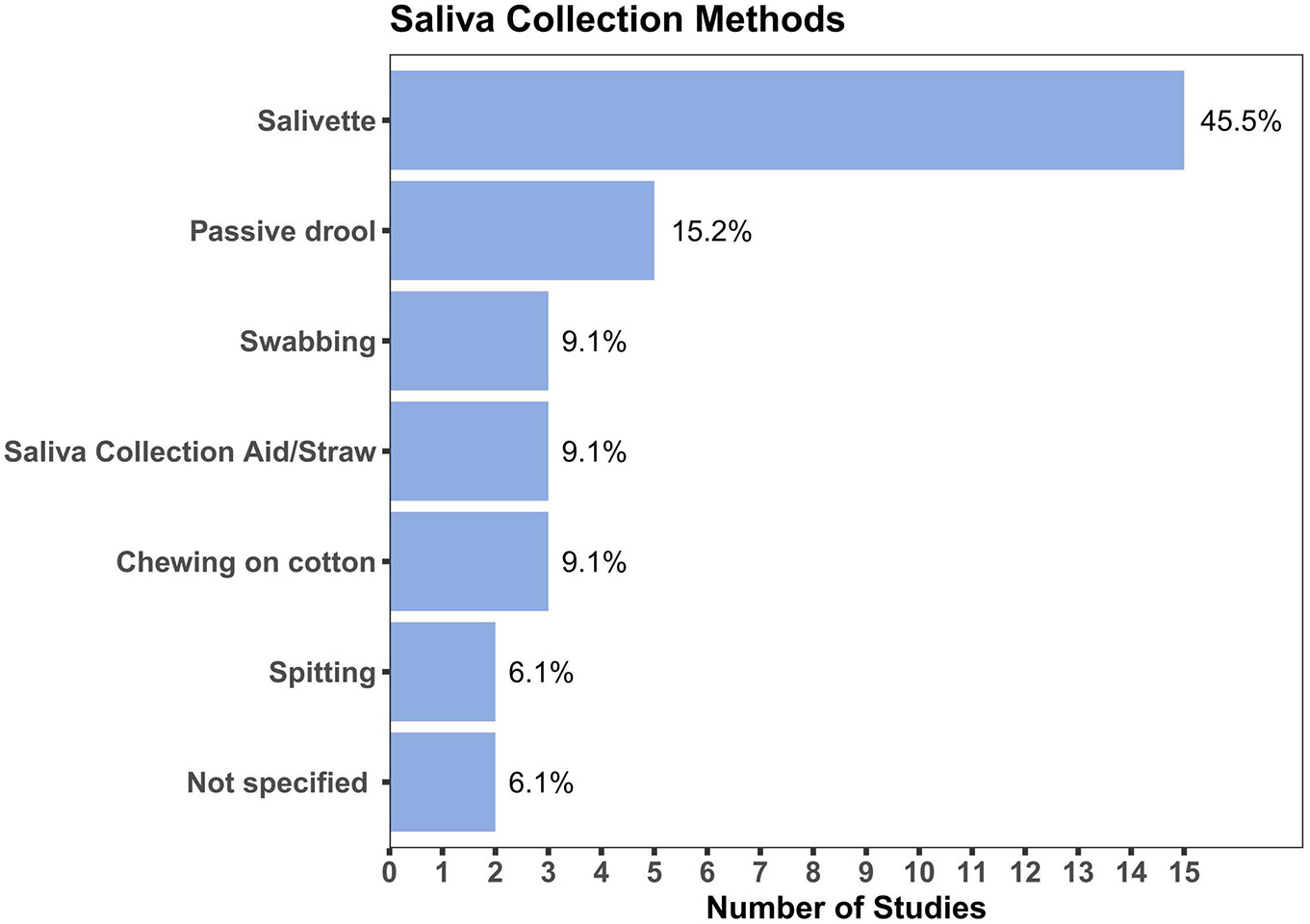
Distribution of saliva collection methods across all included studies demonstrating the majority use Salivette-based methods. N = 4 obtained via personal communication (Personal Communication, Sivan Raz, 2026; Personal Communication, Abigail Beech, 2026; Personal Communication, Heidemarie Laurent, 2026; Personal Communication, Tracy A. Dennis-Tiwary, 2026).

Studies varied substantially in how cortisol was sampled and are outlined in Fig. 3. Two studies did not report methods for collecting saliva and these could not be obtained via personal communication. The largest percentage of studies constrained saliva collection to the afternoon (n = 14), then the morning time (n = 8) (as confirmed by personal communication: Personal Communication, Karen L. Lindsay, 2026), and several other studies used mixed collection times (n = 10) (as confirmed by personal communication: Personal Communication, Kate Keenan, 2026; Personal Communication, Tracy A. Dennis-Tiwary, 2026; Personal Communication, Susannah E. Murphy, 2026) or did not report time of day and this could not be obtained via personal communication (n = 1). Participants across studies were also given varying levels of instruction regarding meals prior to saliva collection: some studies instructed participants to fast to some extent prior to sample collection (n = 15), eat a standardized meal or drink (n = 2), or gave no instructions (n = 14).

Across the 33 included studies, stressor reactivity was quantified in multiple ways. The most common approach was Area Under the Curve (AUC; n = 14) with studies using both AUC with respect to ground (AUCg), which indexes total cortisol output across time, and AUC with respect to increase (AUCi), which captures within-person cortisol output relative to baseline (Pruessner et al., 2003). Of studies using AUC, eight (57.1%) identified significant cortisol responses. Most of studies using ANOVA to assess reactivity also found significant responses (n = 9, 55.6% significant). In contrast, studies using difference scores (n = 4, 25% significant), mixed-effects models (n = 6, 33.3% significant), or *t*-tests (n = 2, 0% significant) were less likely to identify significant cortisol responses. However, these patterns should be interpreted cautiously given the small number of studies within each operationalization category. Further details on operationalization of stressor reactivity across studies can be found in Table 1.

### 3.4. Cortisol reactivity to experimental stress

Across the 33 included studies, 10 distinct experimental stressors were used. Thirteen studies (39.4%) identified a significant cortisol response in some or all participants, 15 (45.5%) found no significant response, and five (15.2%) studies did not report whether cortisol responses were statistically significant.

**Fig. 4.**
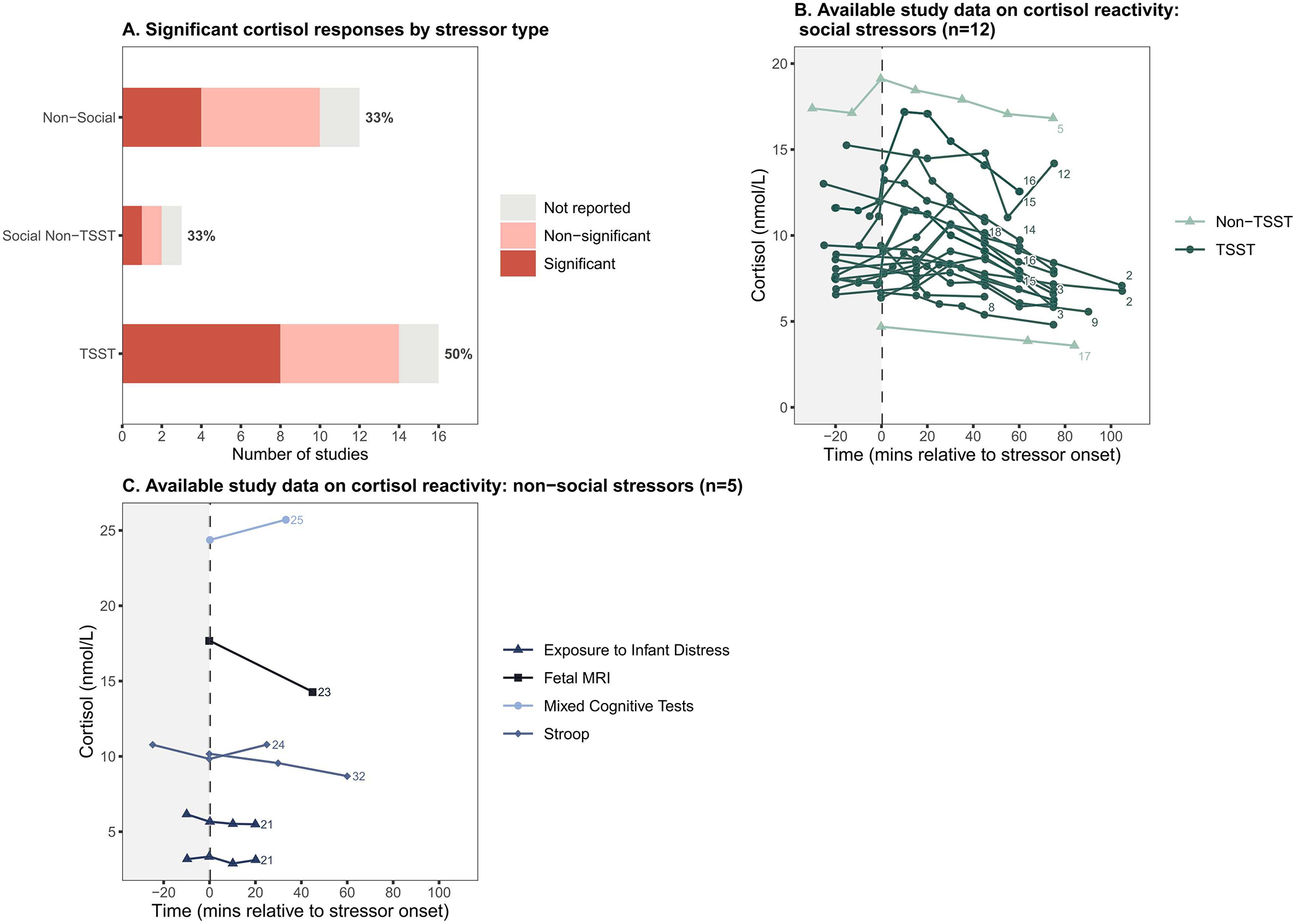
Salivary cortisol responses to social and non-social stressors. The TSST was used most frequently and also most consistently elicited significant cortisol responses among pregnant participants (A). Available data extracted from included papers shows descriptive visualization of cortisol responses to social stressors (B) and non-social stressors (C). In Panel A, “Non-Social” is adjusted to account for confirmed overlapping samples (Personal Communication, Pearl La Marca-Ghaemmaghami, 2026). Numbers corresponding to each line on Panel B and Panel C correspond to study numbers in Table 1. Lines in study Panel B that are not labeled are all from data in study number 19 in Table 1 (Urizar et al., 2019) and are unlabeled because the high number of lines made the citations illegible when added to the graph. Study numbers 15 (Nierop, Bratsikas, Klinkenberg, et al., 2006) and 16 (Nierop et al., 2008) refer to overlapping lines that appear to use the same salivary cortisol data and instead test distinct outcomes, although this could not be confirmed with the authors. The dashed line represents stressor onset; the shaded area represents “baseline” cortisol measurements. Lines above do not include data where participants were grouped by mental health status, as these are shown in Fig. 5. See “Data Analysis” for methods used to extract these data. All data that were reported by the included papers are present here and were converted to nmol/L (the most commonly used unit of cortisol in this review) for interpretability.

**Fig. 5.**
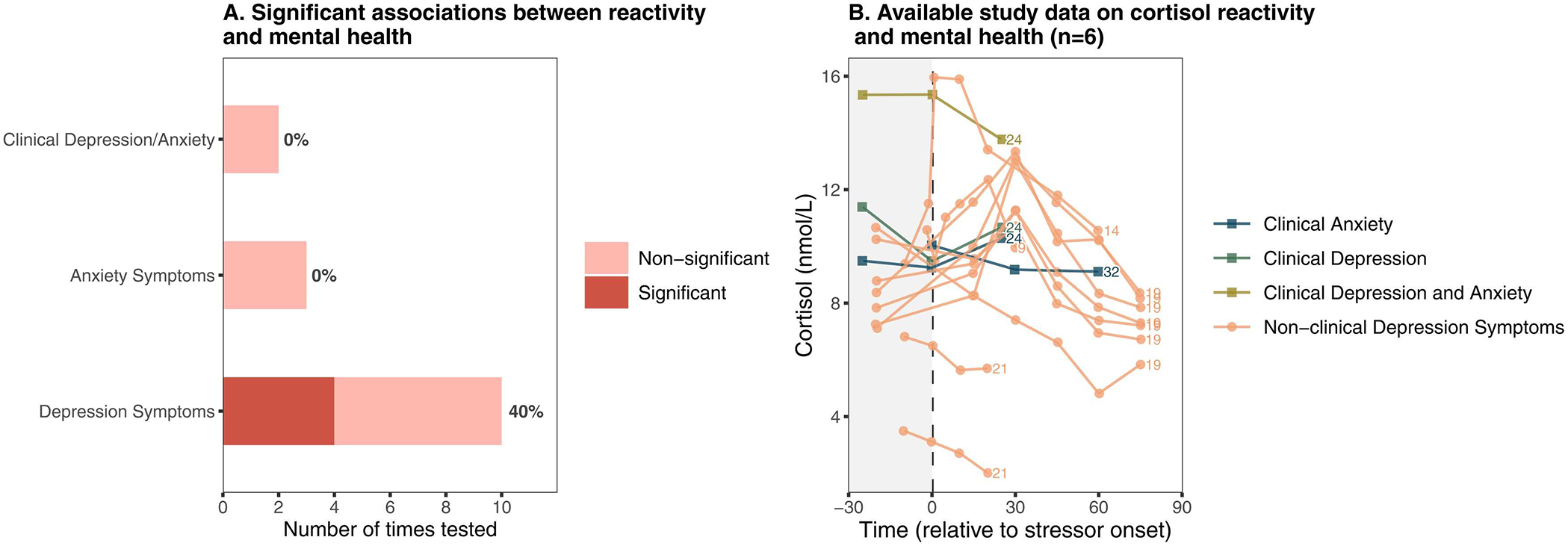
Associations between salivary cortisol reactivity and mental health variables. Associations between cortisol reactivity and depression symptoms were identified in less than half of tests and no associations were identified between reactivity and anxiety symptoms or clinical depression or anxiety (A). Available data extracted from included papers shows descriptive visualization of cortisol responses grouped by mental health status (B). Numbers corresponding to each line on Panel B correspond to study numbers in Table 1. The dashed line represents stressor onset; the shaded area represents “baseline” cortisol measurements.

The following sections review cortisol reactivity findings grouped by stressor type (social versus non-social stressors), and report which studies found associations between cortisol reactivity and mental health, psychological stress, gestational trimester, and race and ethnicity within each stressor category. When studies controlled for rather than directly tested these variables, this is noted in Table 1. Fig. 4, Panel A shows the percentage of significant salivary cortisol responses across stressor category. Fig. 4, Panels B and C show available study data extracted from papers using social stressors (n = 12 available) and non-social stressors (n = 5 available) respectively. Fig. 5, Panel A shows the percentage of significant associations identified between cortisol reactivity and mental health variables. Fig. 5, Panel B in shows available study data extracted from papers that presented data by mental health categories (n = 6 available).

#### 3.4.1. Social stressors (n = 19)

Nineteen studies used stressors with a social component, of which nine studies (47.4%) identified a significant cortisol response in some or all participant groups. Sixteen of the social stressor studies used the Trier Social Stress Test (TSST) as their laboratory stressor (Kirschbaum et al., 1993). The TSST consists of a 5-minute period to prepare for a speech, followed by a 5-minute speech task and a 5-minute math task. These tasks occur in front of two neutral “judges”—one male presenting and one female presenting—who provide no facial or verbal feedback. Of the 16 studies that administered the TSST, eight (50%) observed a significant cortisol response in participants. Two studies did not state whether there was a significant cortisol response to the administered TSST (Dennis-Tiwary et al., 2017; Levendosky et al., 2024). One study used a non-TSST speech and math task with a social-evaluative component in the third trimester and did successfully elicit a significant cortisol response in their sample (de Weerth et al., 2007).

Eight out of 16 TSST studies (50%) made notable protocol modifications such as asking participants to prepare a speech but not deliver it (Beech et al., 2023) or remaining seated throughout the test (Entringer et al., 2010; Lustermans et al., 2024). Additionally, eight TSST protocols used two female judges rather than one female and one male judge (Christian et al., 2013; Ilyumzhinova et al., 2021; Lustermans et al., 2024; Personal Communication, Kate Keenan, 2026; Personal Communication, Kristina M. Deligiannidis, 2026; Personal Communication, Karen L. Lindsay, 2026; Personal Communication, Abigail Beech, 2026; Personal Communication, Pathik D. Wadhwa, 2026). Five studies did not report the presenting gender of the judges and these were not able to be obtained via personal communication. Of the six studies that observed non-significant cortisol reactivity (all during the second and/or third trimesters), all but one modified the TSST by having participants remained seated during the stressor and/or using two female judges (Christian et al., 2013; Deligiannidis et al., 2016; Entringer et al., 2010; Keenan et al., 2014; Lustermans et al., 2024).

The final two social stressor studies used a couple’s conflict discussion using participants in any trimester which did not elicit a significant cortisol response (Roettger et al., 2019), and a cognitive assessment called the Stroop task (Stroop, 1935) with an added a social component (an experimenter prompting subjects to go faster) in the third trimester, in which the researchers did not directly use cortisol reactivity as a measurement and thus did not report effects (Monk et al., 2011).

##### 3.4.1.1. Social stressors: associations with variables of interest.

Three studies reported significant associations between cortisol reactivity to social stressors (all TSST) and depression. In two studies, higher depression symptomatology was associated with greater cortisol reactivity—one in the second and third trimester and one in the first and second trimester (Nierop, Bratsikas, Zimmermann, et al., 2006; Urizar et al., 2019). Beech et al. (2023) found that cortisol reactivity in second and third trimester predicted depressive symptoms one year later, though not concurrent depressive symptoms. Four studies observed null associations between measures of depression and cortisol reactivity to social stressors (Beech et al., 2023; Deligiannidis et al., 2016; Kofman et al., 2019; Levendosky et al., 2024). However, two of these identified interactive effects: Levendosky et al. (2024) found that depressive and post-traumatic stress symptoms moderated the association between intimate partner violence and cortisol output during the TSST in the second trimester, and Kofman et al. (2019) found that lower cortisol reactivity predicted high depressive symptoms only in combination with high maternal neuroticism and low social support from the child’s father, with a marginal negative main effect of depression on reactivity. One study found no association between cortisol reactivity and anxiety in the third trimester (Deligiannidis et al., 2016).

Two studies identified significant associations between psychological stress and cortisol reactivity. High discrimination stress was associated with higher cortisol reactivity in a sample of Black women in their first and second trimesters (Ilyumzhinova et al., 2021) and higher self-efficacy was associated with lower cortisol reactivity in the second and third trimester (Nierop et al., 2008). Four studies observed null associations between psychological stress and cortisol reactivity, including perceived stress or difficult life circumstances in the first and second trimesters (Ilyumzhinova et al., 2021), child maltreatment, post-traumatic stress, or intimate partner violence in the second trimester (Levendosky et al., 2024), perceived stress or affect in the third trimester (Lindsay et al., 2022), and childhood trauma in the third trimester (Deligiannidis et al., 2016).

Evidence for trimester differences in cortisol reactivity to social stressors was mixed. One study found that participants in the third trimester showed significantly higher cortisol reactivity than participants in their second trimester (Nierop, Bratsikas, Klinkenberg, et al., 2006), but a subsequent study by the same group found no significant differences (Nierop et al., 2008). Roettger et al. (2019) similarly identified no differences in cortisol reactivity by gestational age in a sample spanning all trimesters, but the sample size of N = 123 may not have been large enough to detect differences. Notably, Lindsay et al. (2022) observed significant cortisol reactivity to the TSST in third-trimester participants that became non-significant after controlling for gestational age, suggesting gestational age may account for some within-trimester variability in reactivity.

Evidence for racial and ethnic differences in cortisol reactivity to social stressors was limited but notable. Urizar et al. (2019) found that first and second trimester African American women did not mount a significant cortisol response to the TSST, whereas Asian American and non-Hispanic White women did. The same study identified an interaction between ethnicity and depression risk: Latina, Asian American, and non-Hispanic White women with high depression risk showed higher cortisol reactivity, while African American women with high depression risk showed lower cortisol reactivity. One additional study found a marginal, non-significant trend toward lower AUC values in African American women compared to White women in the second trimester, though neither group mounted a significant cortisol response and groups did not differ statistically (Christian et al., 2013). The remaining 17 social-stressor studies did not test racial or ethnic differences, though two studies used exclusively Black American samples in the second and third trimester and first and second trimester respectively (Ilyumzhinova et al., 2021; Keenan et al., 2014).

#### 3.4.2. Non-social stressors (n = 14)

Fourteen studies used stressors without a social component, of which four (28.6%) identified a significant cortisol response in some or all participant groups. Notably, three studies using amniocentesis as a stressor have been confirmed to have overlapping samples (Personal Communication, Pearl La Marca-Ghaemmaghami, 2026); accounting for this, the proportion of independent studies identifying significant cortisol responses is four of twelve (33.3%).

Three non-social stressor studies used variations of a Stroop color-word task in the second and/or third trimesters (Evans et al., 2008; Laurent et al., 2018; Riddle et al., 2023). While all three used the standard Stroop color-word format, they varied in the tasks administered during the cortisol sampling window (i.e., paced breathing). One study also administered an adapted Stroop task presenting pregnancy-specific anxiety-provoking words such as “miscarriage” (DiPietro et al., 2003; Riddle et al., 2023). None of these studies reported a significant cortisol response to the Stroop task.

Four studies used audiovisual clips of infant distress as a stressor. Three presented participants with a 6-minute video of infants under the six months of age crying (Braithwaite et al., 2015; Braithwaite et al., 2016; Murphy et al., 2015), and one used the Baby Cry protocol, which simulates infant crying at varying frequencies (Carbone et al., 2023; Van Anders et al., 2012). Exposure to infant distress did not elicit a significant cortisol response in three of four studies, all of which sampled second and third trimester participants (Braithwaite et al., 2015; Braithwaite et al., 2016; Carbone et al., 2023).

Three studies used amniocentesis—a medical procedure in which amniotic fluid is extracted via a thin needle—as a stressor in the second trimester. A significant salivary cortisol response was observed in one study (Ghaemmaghami et al., 2014) but was not reported in the other two (La Marca-Ghaemmaghami et al., 2013, 2017). As noted above, these three studies share overlapping samples and should not be treated as independent replications (Personal Communication, Pearl La Marca-Ghaemmaghami, 2026).

The remaining four non-social stressor studies used heterogeneous paradigms. Two studies restricted to third trimester participants reported significant cortisol responses: one using a digit-symbol coding test, an arithmetic task, and a stop-signal task with concurrent EEG recording (Fiterman and Raz, 2019), and one using high noise exposure from a nonstress test (NST) device (Turan et al., 2024). Two studies found non-significant cortisol responses: one using cold pressor (hand immersion in ice water) in the third trimester (Kammerer et al., 2002), and one using fetal magnetic resonance imaging in second and third trimester participants (Derntl et al., 2015).

##### 3.4.2.1. Non-social stressors: associations with variables of interest.

Evidence for associations between cortisol reactivity to non-social stressors and mental health was limited and mixed. One study observed that only first and second trimester participants with high depressive symptoms showed cortisol reactivity to infant distress (Murphy et al., 2015). Four additional studies spanning the second and third trimesters found no differences in cortisol reactivity by depressive symptoms (Braithwaite et al., 2015, 2016), or clinical diagnosis of depression and/or anxiety (Evans et al., 2008; Riddle et al., 2023). Two studies also found no association between anxiety symptoms and cortisol reactivity (Ghaemmaghami et al., 2014; Riddle et al., 2023).

Three studies tested associations between cortisol reactivity and psychological stress measures and found no significant effects, including perceived emotional support in the second trimester (La Marca-Ghaemmaghami et al., 2013), psychological stress in the second trimester (Ghaemmaghami et al., 2014), and perceived stress or worries in the third trimester (Riddle et al., 2023).

Two studies found no differences in cortisol reactivity between second and third trimester participants (Braithwaite et al., 2015, 2016). No studies using non-social stressors examined differences in cortisol reactivity by race or ethnicity, representing a notable gap given the racial and ethnic differences observed in the social stressor literature.

## 4. Discussion

### 4.1. Overview of findings

The present scoping review is the first to synthesize the literature on salivary cortisol reactivity to acute laboratory stressors during pregnancy. We identified 33 empirical papers, representing 2418 pregnant participants, published between 2002 and 2024. The HPA axis undergoes substantial neuroendocrine adaptation across gestation, and cortisol reactivity to acute stress is relevant to maternal and offspring outcomes across multiple domains. Despite this, the overall picture that emerges from this literature is one of inconsistency as it pertains to whether studies successfully elicited cortisol responses, and in the associations observed between reactivity and the variables of interest. Fewer than half of the included studies (n = 13, 39.4%) reported significant cortisol responses to the administered stressor. Associations between cortisol reactivity and mental health were detected in fewer than one third of tests (4 of 15, 26.7%), and associations with psychological stress in fewer than one third as well (2 of 7, 28.6%). Gestational trimester and racial and ethnic group were each tested fewer than five times, limiting conclusions, though associations were observed in one of four trimester comparisons and one of two racial and ethnic group comparisons. Substantial variability in how cortisol reactivity was measured and reported across studies complicates the synthesis of this literature further and is discussed in detail below.

### 4.2. Social versus non-social stressors

Stressors with a social-evaluative component were more consistent in eliciting cortisol responses among pregnant participants than those without (47.4% vs. 33.3%). This is consistent with meta-analytic work in non-pregnant populations demonstrating social-evaluative stressors produce both larger and more reliable cortisol responses than non-social stressors (Dickerson and Kemeny, 2004). The relative advantage of social-evaluative stressors may be even more pronounced in pregnancy, given that gestational elevations in basal cortisol and documented challenges activating the HPA axis make eliciting a significant cortisol response particularly challenging in this population (de Weerth and Buitelaar, 2005; Howland et al., 2017).

Nearly half of included studies (n = 16, 48.5%) used the TSST, making it the most common paradigm in this literature. However, half of these studies (n = 8) made modifications to the standard protocol that appeared to attenuate cortisol responses. This pattern mirrors findings in non-pregnant populations, where TSST modifications can substantially reduce cortisol responsivity (for review see, Goodman et al., 2017; Labuschagne et al., 2019). Two specific modifications warrant attention. First, the gender composition of the evaluation panel matters in non-pregnant populations, and mixed-gender presenting panels may be necessary to elicit a cortisol response to the TSST in pregnant samples (Duchesne et al., 2012; Goodman et al., 2017; Labuschagne et al., 2019). Second, posture during the task may influence HPA activation, as salivary cortisol is increased in upright as compared to sitting positions both generally and during the TSST specifically (Hennig et al., 2000; Kurz et al., 2025). Many of the modifications identified in this review were made deliberately to accommodate the comfort and safety of pregnant participants, particularly in later gestation, and such accommodations are understandable and, in some cases, may be necessary. Nevertheless, given the existing challenge of eliciting significant cortisol responses in pregnancy, we recommend that researchers minimize modifications to the standard TSST protocol wherever feasible and clearly document any deviations to facilitate cross-study comparison.

### 4.3. Cortisol reactivity and measures of mental health

Across studies that tested associations between prenatal cortisol reactivity and depression, all that identified significant associations found them in the positive direction. That is, higher depressive symptomology was associated with greater cortisol reactivity (Murphy et al., 2015; Nierop, Bratsikas, Zimmermann, et al., 2006; Urizar et al., 2019). However, fewer than half of studies that tested this relationship found a significant association, and no study identified an association between cortisol reactivity and anxiety. A critical interpretive issue concerns the distinction between subclinical and clinical depression. Studies that identified positive associations with concurrent depression relied on continuous symptom questionnaires and largely excluded participants with a clinical diagnosis of psychiatric conditions (Nierop, Bratsikas, Zimmermann, et al., 2006; Urizar et al., 2019). In contrast, no study that enrolled participants based on a clinical depression diagnosis reported a significant cortisol response (Evans et al., 2008; Monk et al., 2011; Riddle et al., 2023). This pattern could reflect physiologically meaningful differences between subclinical and clinical depression in women— in non-pregnant, female populations, clinical depression is associated with a blunted rather than heightened HPA axis reactivity (Zorn et al., 2017). However, a critical confound complicates this interpretation: all studies using clinically depressed samples also used the Stroop task as the experimental stressor. Given that non-social-evaluative stressors are less reliable in eliciting cortisol responses generally (Dickerson and Kemeny, 2004), it is not possible to determine from the existing literature whether null responses in clinical samples reflected blunted HPA reactivity, an insufficiently potent stressor, or both. Disentangling these explanations is an important target for future research, ideally using social-evaluative stressors in pregnant samples diagnosed with clinical depression.

The association between prenatal cortisol reactivity and postpartum depression warrants particular attention. HPA axis dysregulation during pregnancy is theorized to contribute to postpartum depression risk (Glynn et al., 2013). Accordingly, Beech et al. (2023) found that prenatal cortisol reactivity predicted depressive symptoms one year postpartum—even in the absence of an association between prenatal cortisol reactivity and concurrent depression symptoms. This temporal pattern suggests that cortisol reactivity during pregnancy may index a trajectory of HPA axis functioning that extends beyond the perinatal period. In non-pregnant populations, heightened cortisol reactivity to mild acute stressors without social evaluation was associated with later increases in depressive symptoms (Morris et al., 2012), suggesting a common underlying mechanism. The present evidence points to a complex relationship between cortisol reactivity and mental health during pregnancy that is impacted by symptom severity, stressor type, and timing of assessment that extends from pregnancy into the postpartum period. The interpretation of these associations should also consider the broader context of prenatal depression and anxiety, which co-occurs with factors such as sleep, nutritional deficiencies, poverty, and illness or infection risk that may independently influence cortisol regulation.

### 4.4. Cortisol reactivity and psychological stress

Associations between psychological stress and cortisol reactivity were largely null in the present review, with only two of seven studies reporting significant effects: one finding higher discrimination stress associated with lower reactivity (Ilyumzhinova et al., 2021), and one finding higher self-efficacy associated with lower cortisol reactivity (Nierop et al., 2008). This pattern is broadly consistent with recent reviews showing primarily null associations between prenatal psychological stress and maternal cortisol more generally (Rinne et al., 2023), though maternal exposure to early life adversity appears to more consistently predict cortisol during pregnancy than contemporaneous stress measures (Epstein et al., 2021; Rinne et al., 2023). The null findings are nonetheless somewhat surprising given that in non-pregnant populations, stress exposure across the life course-—including early life adversity, chronic stress, and past-month stress—is associated with blunted cortisol reactivity (Bunea et al., 2017; Creswell et al., 2025; Lam et al., 2019; Lovallo et al., 2012), and protective factors such as social support buffer cortisol responses to acute stress (for review see Hostinar et al., 2014).

Several pregnancy-specific mechanisms may help explain these null associations. First, gestational elevations in basal cortisol may create a ceiling effect that obscures the influence of psychological stress on reactivity. Further, the temporal lag between stress exposure and physiological adaptation may mean that contemporaneous stress measures are poorly timed with HPA axis changes as a result of the stress, especially when the neuroendocrine condition in pregnancy is changing rapidly (Young et al., 2021). Second, pregnancy is also a period of alterations to cognitive processes and brain structure that are hypothesized to support preparation for infant caregiving and may modify how psychological stressors are appraised and processed (Glynn et al., 2004; Kim, 2016; Sejer et al., 2026; Younis et al., 2025). Notably, these changes are not uniformly dampening: enhanced encoding of emotional faces and heightened neurological activation to threat cues during pregnancy suggest that maternal vigilance to social signals is increased (Pearson et al., 2009; Roos et al., 2011). This apparent paradox of attenuated HPA reactivity alongside enhanced threat vigilance may reflect two sides of the same “coin” of physiological adaptations that may serve the maternal-fetal unit. Specifically, protecting against sustained glucocorticoid exposure while maintaining sensitivity to environmental threats. These neural, cognitive, and neuroendocrine adaptations may be important for future research to contextualize HPA axis regulation in pregnancy.

### 4.5. Cortisol reactivity across racial and ethnic group

A central aim of this review was to characterize prenatal cortisol reactivity within racially and ethnically diverse cohorts. Unfortunately, we concluded that the lack of a diverse racial and ethnic composition was one of the most significant gaps in this literature. Twelve of 33 studies (36.4%) did not report race or ethnicity at all, and only two studies directly tested differences in cortisol reactivity by racial or ethnic group. Among the 21 studies that reported these data, the largest proportion of participants identified as White (41.6%), underscoring the degree to which this literature has been generated from majority-White samples. This representational imbalance limits the field's ability to characterize normative cortisol reactivity during pregnancy across diverse populations and is particularly consequential given well-documented racial disparities in perinatal health outcomes.

Despite the limited evidence, a preliminary pattern emerges: Black American women appear to show blunted cortisol reactivity to acute stress during pregnancy compared to women of other racial and ethnic groups (Christian et al., 2013; Keenan et al., 2014; Urizar et al., 2019). Critically, the most specific evidence points to discrimination stress—rather than general psychological stress or stress associated with low income—as a key correlate of this blunted pattern. Ilyumzhinova et al. (2021) found that higher levels of discrimination stress were associated with lower cortisol reactivity in Black women in the first and second trimester, while negative life events associated with economic hardship were not associated with cortisol reactivity. This distinction suggests that racialized stress exposure may be driving HPA axis regulation in this population rather than socioeconomic stress more broadly, though low income and race are deeply confounded in these samples and conclusions remain tentative.

Blunted cortisol reactivity in the context of chronic stress exposure is consistent with a well-established pattern in which sustained HPA axis activation leads to physiological downregulation over time, considered a form of allostatic adaptation to chronic or early life adversity (Koss and Gunnar, 2018). While exposure to stressors is common across racial and ethnic groups, historically minoritized populations (e.g., Black women in the United States) report the highest number of stressors in the year preceding delivery (Lu and Chen, 2004), and flatter diurnal cortisol patterns have been documented in Black American women compared to White American women in non-pregnant cohorts (Adam et al., 2015; Busse et al., 2017; Fuller-Rowell et al., 2012; Hill et al., 2017). Chronic exposure to discrimination and structurally embedded stressors may therefore contribute to changes in HPA axis regulation that could manifest as blunted reactivity in pregnancy—and may in turn help explain the persistent perinatal health disparities between Black and White Americans (Allen et al., 2019; Geronimus, 2001; Ilyumzhinova et al., 2021; Leonard et al., 2019). However, disentangling blunted or hypo-responsivity during pregnancy due to chronic stress exposure from the expected attenuated cortisol reactivity across gestation remains a fundamental methodological challenge that future research must directly address.

### 4.6. Experimental deviations

Despite progress on methodological fronts since de Weerth and Buitelaar’s (2005) foundational review (i.e., larger sample sizes and measurement of stress recovery), substantial heterogeneity in study methods persists. In many ways, this heterogeneity is not surprising: pregnant cohorts can be challenging to recruit and retain as participants may have limited time, may be apprehensive about research procedures, and/or quickly ‘age out’ of gestational timing windows (for review see Frew et al., 2014). Laboratory-based cortisol reactivity paradigms also introduce additional travel and scheduling challenges. Investigators who modify acute stress protocols to improve accessibility likely make reasonable and often necessary accommodations. Nevertheless, the cumulative effect of these deviations is a literature that is difficult to synthesize, especially when attempting to tease out which methodological differences may be driving inconsistencies in the associations between cortisol reactivity and outcomes of interest.

Three domains of methodological variation were particularly notable across included studies: diurnal timing, participant instructions, and how cortisol reactivity was operationalized. The first methodological variation concerns time of day for cortisol sampling, as baseline cortisol levels vary substantially depending on when a laboratory stressor is administered. Cortisol follows a diurnal rhythm such that it is high at wake, peaks in the first ~45 min after wake, and declines across the day. Afternoon testing that allows for adequate time after the midday meal is recommended to help minimize diurnal inter-individual variability (de Weerth and Buitelaar, 2005; Labuschagne et al., 2019). Nevertheless, studies in this review varied widely in sampling time. Where afternoon testing is not feasible, a reasonable alternative to facilitate literature synthesis in the future may be to create balanced groups of participants who complete stressor paradigms early versus late in the day to accommodate challenges in scheduling pregnant participants and report results by timing condition.

The second domain of methodological heterogeneity concerns pre-testing instructions. Cortisol levels are impacted by recent food consumption, smoking, medication use, teeth brushing, and mouth rinsing before saliva sampling (Foley and Kirschbaum, 2010; Kirschbaum et al., 1997). Of the fifteen studies that reported fasting instructions, the required fasting window ranged from 45 min to 8 h, and instructions regarding other pre-testing behaviors were inconsistently reported. For the TSST, fasting for at least 60 min is recommended prior to the laboratory arrival, but up to 2 h may be needed for cortisol levels to return to baseline after mid-day meal consumption (Gibson et al., 1999; Narvaez Linares et al., 2020). In non-pregnant adults, standardized glucose intake (e.g., a glucose beverage) may improve cortisol reactivity (Kördel et al., 2025), but it is unclear if this standardization would help in pregnancy. Standardizing these instructions across studies—and reporting them transparently—would substantially improve comparability.

The third domain of methodological heterogeneity involves the operationalization of cortisol reactivity. Studies used a range of analytic approaches, including repeated measures ANOVA, AUCg, AUCi, difference scores, *t*-tests, and mixed-effects models. These are not interchangeable: AUCg and AUCi capture different physiological information (Pruessner et al., 2003). Likewise, total output measures and change-from-baseline measures in non-pregnant cohorts may index distinct components of stress responsivity even within the same sample (Khoury et al., 2015). Researchers using different indices may therefore be examining different physiological phenomena while appearing to study the same construct. As a practical step toward improving cross-study comparability, future studies should report means and standard deviations of relevant information at each sampling timepoint alongside their primary reactivity outcome. Specifically, studies should report means and standard deviations of cortisol time in minutes since stressor onset at each sampling time point. Only four studies in the present review did so, severely limiting the descriptive synthesis possible in this scoping review and precluding future meta-analytic work.

Remote stress paradigms are one emerging methodological strategy that may increase accessibility of acute stress reactivity paradigms without requiring the protocol modifications that appear to attenuate cortisol responses in pregnant samples. For example, remote TSST paradigms have been validated in youth (Gunnar et al., 2021) and adult (Meier et al., 2022) populations, though validation of remote TSST paradigms in pregnant populations remains an important prerequisite. Fundamentally, the core challenges identified in the present review (time-of-day variability, inconsistent pre-testing protocols, and heterogeneous operationalization of reactivity) were raised by de Weerth and Buitelaar over 20 years ago and underscore the persistence of these issues and the urgency of addressing them as this literature continues to grow.

### 4.7. Recommendations for future research

The findings of this scoping review point to four interconnected recommendations for future research that can be organized around stressor validation, gestational timing, population diversity, and methodological standardization.

First, the most widely used stressors in this literature—the TSST and the Stroop task—were developed and validated in non-pregnant populations. The methodological accommodations documented across the included studies reflect genuine efforts to adapt these paradigms for pregnant participants, for whom standard protocols might be uncomfortable. However, the downstream consequence is a fragmented literature in which modifications vary across studies in ways that are difficult to account for analytically or even narratively. Foundational validation work establishing how widely used stress paradigms perform in pregnant populations and what modifications are acceptable without meaningfully attenuating cortisol responses would provide the field with an empirical basis for standardization and substantially reduce methodological heterogeneity going forward.

Second, characterizing how cortisol reactivity changes across gestation remains an unresolved and important question. Most included studies did not report cortisol responses separately by trimester or gestational age windows, preventing a robust characterization of normative change across pregnancy. Of the four studies that directly compared second and third trimester participants, only one found significantly greater reactivity in the third trimester. If we were to speculatively conclude anything based on this very limited evidence, this may suggest that cortisol reactivity attenuation *may* be somewhat stable after the first trimester. We caution against this interpretation, however, until future studies can robustly test this associations in larger samples. Future studies that test trimester differences longitudinally and within individuals where possible would greatly benefit the literature. Where repeat longitudinal stressor paradigms are not possible, we encourage future studies to report trimester differences between individuals rather than simply controlling for gestational age. Moreover, future studies should report supplemental tables of cortisol outcomes by trimester and mean gestational age within each trimester to help future researchers contextualize existing data, make more robust conclusions, and facilitate future synthesis.

Third, the demographic scope of this literature requires urgent expansion. Only one third of included studies reported household income, none examined associations between income and cortisol reactivity, and only two of 33 studies directly reported cortisol reactivity differences by racial or ethnic group despite over half reporting these demographics. Moreover, the vast majority of the world's pregnancies occur outside of the United States and Europe, but these geographic locations together accounted for 87.9% of included studies. For context, in 2012, 89% of the world's 213 million pregnancies occurred in low- and middle-income countries, with over half in Asia and 25% in Africa (Sedgh et al., 2014). This geographic and demographic concentration severely limits the generalizability of current findings and leaves critical questions entirely unaddressed about how stress physiology during pregnancy varies across diverse populations, environmental pressures, and structural contexts. Expanding this literature to include more racially, socioeconomically, and geographically diverse samples is not only scientifically important but essential for understanding how stress reactivity during pregnancy contributes to disparities in maternal and child health outcomes (Christian et al., 2013).

Fourth, as discussed in Section 4.6, methodological standardization in sampling timing, pre-testing instructions, and operationalization of cortisol reactivity would substantially improve the comparability and synthesizability of future work. We recommend that all future studies report means and standard deviations of cortisol levels and time in minutes since stressor onset at each sampling time point as a minimum standard for transparency and an essential practice for any future meta-analytic work in this area.

### 4.8. Strengths and limitations

Several limitations of this review should be noted. First, as a scoping review, we are unable to make quantitative claims about the magnitude of cortisol reactivity during pregnancy. Though this is by design, it means that effect sizes, pooled estimates, and formal tests of heterogeneity remain outstanding questions for future meta-analytic work. The limited reporting of individual cortisol levels across time points in included studies further constrained our descriptive synthesis, though we attempted to mitigate this by digitizing cortisol values from published figures where possible. Second, the scope of this review was limited to peak cortisol reactivity and did not include steepness of cortisol increase, speed of increase, or speed of recovery following stressor offset, though these are distinct indices of HPA axis function outside of pregnancy, and may be linked to different downstream health outcomes (Degering et al., 2023; M. Gunnar and Quevedo, 2007; Young et al., 2021). Third, several studies reported significant associations between predictors and/or outcomes and baseline cortisol but not reactivity, which fell outside the scope of this review but may be relevant to whether and how participants mounted cortisol responses to administered stressors. Fourth, when included studies did not report key methodological details (e.g., saliva collection methods, time of day for sampling, potential sample overlap with other published studies, or gender composition of TSST evaluation panels), we contacted authors directly. However, we did not receive responses to six of these inquiries, leaving some methodological uncertainty unresolved. Finally, our search strategy may have missed studies in which a cognitive challenge or experimental task functioned as a stressor but was not described using the terms “stress,” “stressor,” or “stressors” in the title, abstract, or database-assigned controlled vocabulary terms. Studies of this kind would not have been captured by our search, and the true scope of the cortisol reactivity in pregnancy literature may be modestly broader than what is represented here. Nevertheless, we did capture 10 different experimental stressor paradigms that ranged from standard psychological stress lab tasks to clinical exposures such as amniocentesis, strengthening our confidence in how stress reactivity in pregnancy has been captured in the current literature.

This review also has several notable strengths. It is the first scoping review to synthesize the literature on salivary cortisol reactivity to acute experimental stressors during pregnancy, filling a gap that two prior narrative reviews called for (Christian, 2012; de Weerth and Buitelaar, 2005). By restricting inclusion to salivary cortisol, we were able to more directly compare cortisol levels across studies with respect to stressor onset in stressor paradigms that are less invasive and more accessible than blood draws to measure cortisol reactivity. This review extended prior work by characterizing experimental paradigms, including the distinction between social and non-social stressors, and by synthesizing findings with respect to participant demographics, mental health, and psychological variables, directly addressing gaps identified by Christian (2012). The breadth of the search, which screened over 1200 studies and spanned more than two decades of research, provides confidence that the literature has been comprehensively characterized. Together, these features position this review as a foundation for the next generation of more targeted, quantitative synthesis of prenatal cortisol reactivity.

## 5. Conclusion

This scoping review mapped 33 empirical studies representing over two decades of research on salivary cortisol reactivity to acute laboratory stressors during pregnancy. The literature reveals an active field that has yet to coalesce around shared methodological standards: fewer than half of studies successfully elicited a cortisol response, and substantial heterogeneity in experimental paradigms, sampling protocols, and analytic approaches limits what can be concluded from the existing evidence. Stressors with social-evaluative components were more reliable in activating the HPA axis than non-social stressors, and preliminary evidence suggests that depressive symptomatology and racialized stress exposure are meaningfully associated with cortisol reactivity during pregnancy, though both relationships require replication in more diverse samples using rigorous and standardized methods. We also call for studies of cortisol reactivity in pregnant populations that reflect where the global majority of pregnancies occur. The persistent methodological challenges documented here, many of which were identified over 20 years ago, underscore the need for foundational validation work and reporting standards specific to pregnant populations. At minimum, we recommend that future studies report mean cortisol levels and standard deviations at each sampling time point, the time in minutes since stressor onset at each sample, demographic differences in stress reactivity, and the gestational age window of participants— a standard that would substantially improve cross-study comparability and lay the groundwork for future meta-analytic synthesis. By comprehensively characterizing the scope and structure of this literature, this review provides the foundation necessary for future analyses to move the field toward a more precise, generalizable, and equitable understanding of stress physiology during human pregnancy.

## Figures and Tables

**Table 1:** Synthesis of included studies (n=33), organized by type of stressor. GA – Gestational age; AUCi – Area Under the Curve with respect to increase; AUCg – Area Under the Curve with respect to ground. Data in this table are reported directly from each individual study and are not otherwise synthesized (i.e., if a paper reported percentages rather than counts, this value was not converted to match other papers). Given this review’s focus on characteristics of the pregnant individual as it relates to cortisol reactivity, variables listed here reflect only mental health measurements, psychological stress measurements, gestational age characterizations, and/or race and ethnicity categories. Additionally, some papers contained non-pregnant comparison groups but results from these groups are excluded, as they are not within the scope of the present review.

	Study	Subjects	Trimester	Stressor	# of samples	Operationalization and Analysis of Reactivity	Subgroups	Cortisol Response to Stressor	Associations with Variables of Interest	Additional Cortisol Findings
	**Social Stressors**									
1	Beech et al., (2023)	**N = 33**; 21 White, 6 Asian, 4 Black/African American, 5 Hispanic, 0 American Indian/Native American, 2 Middle East and North Africa/Arab, 2 Completed High School or less	**2nd and 3rd trimester**; Range 16–39 weeks, Mean GA: 29.5 (SD= 5.90)	**Modified TSST** - participants completed mental arithmetic and prepared a speech but were told they had been randomly excused from presenting it; female judges only	**4**	AUCi; AUCi= AUCg – Cort2(time interval 2 + time interval 3)	No groups within perinatal participants	Cortisol reactivity to TSST was positive for both the perinatal and nulliparous groups	**Mental Health Measure** - Higher cortisol reactivity at Time 1 (between January 2018 and January 2020) predicted higher depressive symptoms at Time 2 (April 2020), controlling for depressive symptoms at Time 1. Cortisol reactivity at Time 1 was not associated with concurrent depressive symptoms	There were no differences in cortisol reactivity between perinatal and nulliparous participants
2	Christian et al., (2013)	**N = 39**; 20 White, 19 Black/African American, 14 Completed High School or Less; 17 < $15,000, 12 $15,000-$29,999, 10 $30,000	**2nd trimester**; Range: 21–24 weeks, Mean GA: N/A (SD=N/A)	**Modified TSST** - the audience was composed of two female evaluators, one African American and one white	**7**	Linear mixed models assessed differences in stress responses (IL-6, salivary cortisol, and PANAS affect) across race and pregnancy, and the association between cortisol and IL-6 change was evaluated using the area under the curve (AUC) for salivary cortisol from 25 min pre- to 90 min post-stressor, calculated via the trapezoidal rule	**2 Groups: Pregnant White** and **Pregnant African American**	Salivary cortisol was significantly lower than baseline immediately post-stressor and at 30, 45, 60, and 90 min post-stressor. There were no significant increases from baseline in cortisol in any of the groups.	No relevant variables	At baseline, cortisol was significantly higher in pregnant women than in nonpregnant women. A non-significant trend was seen for lower baseline cortisol among African Americans versus Whites. There were no significant differences in estimated slopes for linear change in salivary cortisol from 25 min pre-stressor to 90 min poststressor.
3	Deligiannidis et al., (2016)	**N = 44**; 39 White, 6 Hispanic/LatinX, 5 Other; 6 Completed High School or Less	**3rd trimester**; Range: 28–33 weeks, Mean GA: 31.3 (SD=2.3)	**Modified TSST** - two female judges	**6**	Generalized estimating equation (GEE) methods to control for the correlation within the subjects at the six time points during the TSST. AUCi was calculated using a trapezoidal formula for salivary cortisol at all six time points	**2 Groups: Low-Risk Healthy Comparison** and **At-Risk for Postpartum depression**	Average cortisol values decreased in both groups over time. The majority of subjects had a negative AUCi	**Mental Health Measure** - Healthy pregnant women and those at-risk of PPD had no detectable difference in salivary cortisol response to psychosocial stress induced by the TSST. GEE models examining longitudinal changes in cortisol did not differ significantly by Edinburgh Postnatal Depression Scale score. State-Trait Anxiety Inventory total score also did not affect cortisol concentration over time and did not differ by group; **Psychosocial Stress Measure** - GEE models examining longitudinal changes in cortisol did not differ significantly by Childhood Trauma Questionnaire score	There was no correlation between Edinburgh Postnatal Depression Scale score and baseline cortisol
4	Dennis-Tiwary et al., (2017)	**N = 29**; 15 White, 7 Asian, 1 Black/African American, 6 Hispanic/LatinX, 1 American Indian/Native American, 2 Mixed, 3 Other; Mean Income=$209,180 (SD=$232,990), Income Range= $17,000 - $1,000,000	**2nd and 3rd trimester**; Range: N/A, Mean GA: 22.44 weeks (SD= 2.43)	**TSST**	**3**	Slope, AUCi = indicates change in concentration across a specific period of time (in the present study, the three time points within each context) indicating the magnitude and direction of change. Thus, a negative value reflects a decrease over time whereas a positive value reflects an increase over time. Weeks pregnant upon entry into the study was used as a covariate given the link between gestational period and cortisol level	**2 Groups: Placebo Training (PT) App** and **Attention Bias Modification Training (ABMT) App**	Could not determine based on reported results	No relevant variables	Cortisol secretion over the course of lab-based stressors was reduced in the ABMT versus PT group. Reduced lab AUCi was associated with lower levels of vigilance toward threat. Reduced home AUCi (from the first morning sample to the evening sample) was associated with less post-training anxiety
5	de Weerth et al., 2007	**N = 120**; 120 White; 24.4% Completed High School or Less	**3rd trimester**; Range: N/A, Mean GA: 32.8 weeks (SD=1.1)	**Experimental psychosocial stressor** - including a Public Speaking and Mental Arithmetic Test	**7**	The AUC of the 4 dependent variables were normally distributed, with the exception of the AUCi for cortisol. In order to normalise this variable, the square root was calculated, and used for further analyses. The cortisol AUCi was significantly positively correlated to the cortisol AUCg. Correlations between different variables and including AUCi, are partial correlations controlling for the first basal value of the variable(s) in question as the pre-stress levels could influence the magnitude of the reaction.	No groups within perinatal participants	All the stress values are significantly higher than all the resting values, with two exceptions - the third stress value and first pre-stress resting value; 49.5% of the pregnant women showed no cortisol reaction	No relevant variables	N/A
6	Entringer et al., (2010)	**N = 148**; 14.5 + /− 2.5 Completed High School or Less	**2nd and 3rd trimester**; Range: 17–31 weeks, Mean GA at first assesment: 16.6 (SD=0.14), Mean GA at second assessment: 31.5 (SD=0.12)	**Modified TSST** - thirty minutes prior to the onset of the TSST, participants were seated in a comfortable armchair in a recumbent position and were instrumented with Dinamap vital signs monitors for automated assessment of HR and blood pressure. Participants remained seated in this position and in the same room during the whole procedure	**6**	Hierarchical Linear Modeling (HLM), linear and quadratic slopes; Time was centered at baseline so that the intercept represented the mean cortisol levels just prior to the start of the TSST. Used precise measures of time of day for sample collection and gestational age at testing rather than nominal estimates of assessment intervals in order to control for variability in the timing of collections or stage of pregnancy.	No groups within perinatal participants	Exposure to TSST did not produce a significant increase salivary cortisol concentration at either of the two assessments in the pregnant group.	No relevant variables	A significant increase in cortisol concentrations was observed in the first hour after awakening, and there was also a quadratic effect. Furthermore, from awakening to 20:00 h, cortisol concentrations decreased significantly reflected by the linear day time slope. These results suggest that during pregnancy, the diurnal cortisol rhythmicity (increase in response to awakening and a general decrease over the course of the day) is preserved. The diurnal cortisol rhythm over the course of the day did not change significantly as gestation advanced
7	Ilyumzhinova et al., (2021)	**N = 100**; 100 Black/African American; receipt of public assistance (i.e., Medicaid insurance) part of inclusion criteria	**1st and 2nd trimester**; Range: 10–17 weeks, Mean GA: N/A (SD=N/A)	**Modified TSST** - participants were brought into a room where two female judges (one Black and one non-Black) sat at a table. The judges played recorded instructions to the participant for the speech task	**5**	AUCg which captures the magnitude of change regardless of direction (i.e., increase or decrease), was calculated for cortisol.	No groups within perinatal participants	Participants who experienced higher levels of discrimination stress had no increase in cortisol following the TSST. Participants who experienced lower levels of discrimination stress had increased levels of cortisol 20 min following the TSST.	**Psychosocial Stress Measure** - Discrimination, but not perceived stress or difficult life circumstances, explained variance in cortisol response to the TSST. Specifically, discrimination stress was negatively associated with cortisol response. Participants who had lower levels of discrimination stress on average showed a pattern of increased cortisol levels 20 min following completion of the TSST. The difference between the two groups in peak response at 20 min following the TSST was statistically significant.	N/A
8	Keenan et al., (2014)	**N = 64**; 64 Black/African American; Eligibility included Medicaid insurance or Medicaid eligible	**2nd and 3rd trimester**; Range: 24–30 weeks, Mean GA: N/A (SD=N/A)	**Modified TSST** - two female judges, one White and one non-White (usually Black)	**3**	Repeated measures ANOVA (pre- and post TSST). Time of day was controlled in analyses involving cortisol levels at baseline	**2 Groups: Placebo** and **Active Supplement** (taking 450 mg of docosahexaenoic acid (DHA) per Day)	Levels for women receiving placebo were char_acterized by a relatively steep decline, whereas levels for women receiving omega-3 supplement evidenced a slight increase in response to the stressor, on average, followed by decline during the period of recovery	No relevant variables	Time of day was significantly associated with initial cortisol levels at baseline but not at 24 and 30 weeks of gestation. Cortisol levels on arrival to the laboratory were similar for the two groups at baseline but diverged over time such that by 30 weeks of gestation, women who received placebo had levels that were on average 20% higher than women receiving supplement
9	Kofman et al., (2019)	**N = 49**; 14 White, 5 Asian, 28 Hispanic/LatinX; 13 Completed High School or Less; 21% extremely low income, 9% very low income, 6% low income, 2% median income, 9% above median income	**1st, 2nd, and 3rd trimester**; Range: 9–30 weeks, Mean GA: 21.25 (SD=5.11)	**TSST**	**8 +**	Overall cortisol secretion for the TSST = AUCg. Maximum cortisol response to the TSST = (max value - [−2min sample]). Mean cortisol response to the TSST = ([+1, +5, +10, +20, +30 min samples]/5 – [−2 min sample]). GA was controlled for in all relevant analyses	**2 Groups: High Depressive Symptoms** and **Low Depressive Symptoms**	Pre-TSST cortisol concentrations were higher than cortisol 1 min after the TSST for women high in depressive symptoms. No discernable change in cortisol was observed for women low in depressive symptoms.	**Mental Health Measure** - No discernable change in cortisol was observed for women low in depressive symptoms	High depressive symptom women's baseline cortisol was elevated and on average higher than the saliva sample obtained immediately after the TSST. Women high in depressive symptoms had a flatter cortisol awakening response compared to those with low depressive symptoms. More perceived social support from others was marginally associated with higher Cortisol Awakening Response AUCg.
10	Levendosky et al., (2024)	**N = 255**; 123 White, 4 Asian, 84 Black/African American, 1 American Indian/Native American, 26 Mixed, 16 Other; 136 Completed High School or Less	**2nd trimester**; Range: 15–18 weeks, Mean GA: 16.42 (SD=1.15)	**TSST**	**4**	AUCg	No groups within perinatal participants	N/A - did not specifically state whether there was a change in cortisol as a response to stressor	**Mental Health Measure & Psychosocial Stress Measure** - The main efects of IPV, post-traumatic stress symptoms (PTSS) and depressive symptoms on cortisol AUCg were not statistically signifcant, but the interaction between IPV and depressive symptoms was. At low levels of depressive symptoms, the association between intimate partner violence (IPV) and AUCg was negative; at moderate levels, the association was not signifcant, and at high levels, the association between IPV and AUCg was positive. The effects of IPV on cortisol AUCg were not signifcant at low and moderate levels of PTSS, but there was a positive association at very high levels	Cortisol AUCg was not directly associated with child maltreatment, depressive symptoms, PTSS, and IPV, but was only negatively correlated with the number of times a woman had previously given birth.
11	Lindsay et al., (2022)	**N = 33**; 33 Hispanic/LatinX	**3rd trimester**; Range: 28–32 weeks, Mean GA: 30.2 (SD=1.3)	**Modified TSST** - two female judges	**7**	AUCg - differences in within-person cortisol AUCg between study visits were assessed using paired sample *t*-tests and by repeated-measures ANOVA adjusting for gestational age. Given that not all individuals respond equally to the TSST in terms of stress reactivity, participants were classified as high or less cortisol reactors based on a median split of cortisol AUCg on visit 2	No groups within perinatal participants	Salivary cortisol AUC was significantly higher after acute stress compared to the non-stress control condition, however, after adjusting for gestational age, the difference between visits in cortisol AUC was no longer significant	**Psychosocial Stress Measure** - On both study visits, the measure of physiological stress (cortisol AUC) was not found to correlate with indices of baseline psychological states (perceived stress, positive affect, negative affect at visit 1), or with psychological stress reactivity to the TSST (change in negative affect score on visit 2).	On both study visits, cortisol AUC was not found to correlate with indices of baseline psychological states (perceived stress, positive affect, negative affect at visit 1), or with psychological stress reactivity to the TSST (change in negative affect score on visit 2).
12	Lustermans et al., (2024)	**N = 110**	**3rd trimester**; Range: 29–36 weeks, Mean GA: 32.28 (SD=1.54)	**Modified TSST** - participants were seated instead of standing (advanced pregnancy) and the two judges, both of whom were female, joined through a video call on a large screen (COVID-19 regulations). After the completion of the math task, participants were told to wait alone for five minutes while the judges discussed their performance in private. Then, the researcher came back to tell participants the judges had positively evaluated their performance	**5**	Preliminary analyses were performed to assess group comparisons between Stress Group and Control Group on physiological stress response, using independent samples *t*-tests if they were normally distributed, and Mann–Whitney U tests if they were not	**2 Groups: Control** (CG) and **Stress** (SG; participated in the TSST)	Detected a small increase of salivary cortisol after stressor onset in the SG, but was not a significant difference from prior to the stressor	No relevant variables	A significant difference in the salivary cortisol concentration change between T1 and T2 between the SG and the CG was detected. The T2-T3 saliva sample showed a decrease in cortisol concentrations in both groups. Further inspection of the design revealed that this may have been due to the timing of this specific sample.
13	Monk et al., (2011)	**N = 113**; 60% Latina, 19% White, 5% Asian, 10% Black/African American, 6% Mixed	**3rd trimester**; Range: 37–43 weeks, Mean GA:39 (SD=1)	**Stroop task** - participants were required to push keys that corresponded to the correct color responses as fast as possible. If responses were incorrect or too slow, the computer displayed the message “incorrect” on the screen. The experimenter also prompted participants to work faster throughout the task. This was followed by a paced breathing task where participants alternated among 30 s periods of breathing at a faster than normal rate (approximately 30 breaths per minute), a slower than normal rate (about 10 breaths per minute), and at an approximately normal rate (20 breaths per minute)	**3**	Used baseline cortisol, as opposed to a reactivity measure. Controlled for session start time variation in analyses	**4 Groups: Control** (if no current Axis I Diagnosis), **Depressed** (current major depressive disorder and/or dysthymia), **Anxiety** (current social phobia, a simple phobia, generalized anxiety disorder, or agoraphobia without panic disorder), and **Comorbid** (curent depression and an anxiety disorder, including Post Traumatic Stress Disorder)	N/A - did not use cortisol as a reactivity measure	No relevant variables	Greater maternal cortisol at baseline was associated with overall higher fetal heart rate across all periods. Pregnant women co-morbid for depression and anxiety had higher resting cortisol than women with depression only, an anxiety disorder, or who were controls.
14	Nierop et al., 2006a	**N = 57**; 22 Completed High School or Less	**2nd and 3rd trimester**; Range: 13–31 weeks, Gestational Ages reported by subgroup, see column G	**TSST**	**8 +**	ANOVA with repeated measures; AUCi, AUCg. Possible effects of weeks of pregnancy on cortisol stress reactivity were controlled by conducting analyses of covariance (ANCOVA)	**2 Groups: Probable Noncases** (those scoring 9 and below on Edinburgh Postnatal Depression Scale; Mean GA: 22.45 (SD=6.53)) and **Probable Cases** (those scoring 10 or above on Edinburgh Postnatal Depression Scale; Mean GA: 21.06 (SD=6.84))	The TSST resulted in a significant increase in salivary cortisol	**Mental Health Measure** - The cortisol response to the TSST during pregnancy was significantly higher in the probable case group compared with the probable noncase group without depressive symptoms during early puerperium	N/A
15	Nierop et al., 2006b	**N** = **60**	**2nd and 3rd trimester**; Range: 13–31 weeks, Mean GA: N/A (SD=N/A)	**TSST**	**8 +**	Two-way ANOVA with repeated measurement; AUCi	**3 Groups: Nonpregnant, 2nd Trimester Pregnant** (13–18 weeks), and **3rd Trimester Pregnant** (26–31 weeks)	Stress protocol induced significant increases in cortisol levels and revealed a significant time by group (trimester) interaction	**Trimester** - Significant differences in cortisol levels post-stressor between third-trimester and second-trimester pregnant women	Significantly higher baseline levels of cortisol in third-trimester compared with second-trimester pregnant women and nonpregnant women. AUCIncrease showed no significant differences between groups, indicating that pregnant women show the same cortisol responses caused by psy_chosocial stress compared with women in the follicular phase of their menstrual cycle
16	Nierop et al., (2008)	**N = 60**	**2nd and 3rd trimester**; Range: 13–31 weeks, Mean GA: N/A (SD=N/A)	**TSST**	**8 +**	AUCi; Repeated measure ANOVA across the two trimester groups	**2 Groups: 2nd Trimester Pregnant** (13–18 weeks) and **3rd Trimester Pregnant** (26–31 weeks)	The stress protocol induced significant increases in cortisol levels. AUCi showed no significant differences between the two groups	**Psychosocial Stress Measure** & **Trimester** - Higher self-efficacy, but not trimester, predicted lower cortisol stress reactivity of borderline significance	N/A
17	Roettger et al., (2019)	**N = 123**; 92% White	**1st, 2nd, and 3rd trimester**; Range: 12–32 weeks, Mean GA: 22.37 (SD=4.98)	**Conflict Discussion** Task - couples were asked to discuss three problems in their relationship in a 12-min videotaped interaction. These problem areas were chosen by research assistants based on items each partner had previously rated highly on a list of potentially conflictual topics (e. g., couple relationship difficulties, financial strain)	**3**	The difference in cortisol level at t1 from t0 (t1-t0) and reflects maternal cortisol change immediately following the couple's discussion of relationship stressors	No groups within perinatal participants	Mean cortisol levels across the entire sample declined across the three assessment timepoints	**Trimester** - No significant moderation patterns of gestational age with respect to cortisol reactivity and recovery were found	Among mothers with lower depressive symptoms, motherrated overall child health was consistently high, increasing only slightly with increasing levels of reactivity. In contrast, among mothers with higher levels of depressive symptoms, motherrated overall child health was lower when cortisol reactivity was also lower. Maternal prenatal depressive symptoms did not moderate the associations between cortisol reactivity and maternal reports of specific child health problems
18	Sun et al., 2023	**N** = **63**	**1st and 2nd trimester**; Range: 8–14 weeks, Mean GA: 11 (SD=2)	**TSST**	**5**	Salivary cortisol was used as the dependent variable in a linear model to analyze the differences of pre-post changes in virtual reality environments of middle and high green space groups versus those in non-green space group. The mixed model for repeated measures was applied for salivary cortisol levels.	**3 Groups: Non-Exposure to Green Space, Medium Exposure to Green Space**, and **High Exposure to Green Space**	Salivary cortisol increased significantly for all groups after the TSST	No relevant variables	Participants experiencing green space environments during the recovery period had consistently greater decreases of salivary cortisol, especially for the high green space group
19	Urizar et al., (2019)	**N = 34**; 5 White, 5 Asian, 11 Black/African American, 13 Hispanic/LatinX; 17 Completed High School or Less	**1st and 2nd trimester**; Range: 8–25 weeks, Mean GA: 17.2 (SD=4.8)	**TSST**	**6**	A mixed effect linear model was used to test for possible time and group effects of depression risk and ethnicity on salivary cortisol patterns over the six study time points. Exploratory MANOVA analyses were performed to identify factors that could help explain any observed group differences in cortisol responses to the TSST. Pearson partial correlations were then conducted post hoc to determine if any of these factors were associated with cortisol patterns among these groups while controlling for gestational age, parity, and education	**2 Groups: High Risk for Depression** (met Major Depressive Episode criteria sometime in lifetime, not including the past two weeks) and **Low Risk for Depression** (never met Major Depressive Episode criteria in lifetime)	TSST produced a significant biological stress response among participants	**Mental Health Measure** - Women in the high depression risk group showed greater cortisol reactivity to the TSST and a steeper recovery following the stressor than women in the low depression risk group. Neither levels of dispositional optimism, nor levels of spirituality, as coping resources were associated with cortisol patterns by depression group; **Racial/Ethnic Group** - Asian American and non-Hispanic White women (combined as one group) showed greater cortisol reactivity to the TSST and higher cortisol levels overall than African American women. As a group, African American women did not show any elevation of cortisol in response to the TSST. Latina women at high depression risk demonstrated greater cortisol reactivity and a steeper cortisol recovery to the TSST than Latinas at low depression risk. Similarly, Asian American and non-Hispanic White women who were at high depression risk had greater cortisol reactivity than those at low depression risk. Finally, African American women at high depression risk showed lower cortisol levels overall than African American women at low depression risk	Results demonstrated that women at high depression risk reported feeling more stressed during the speech task of the TSST than women at low depression risk, resulting in lower cortisol reactivity to the TSST for those in the high depression risk group. women at high depression risk appeared to experience more discouragement or frustration during the math task than women at low depression risk. These behavioral responses to the TSST were not associated with observed group differences in cortisol patterns by depression risk group
	**Non-Social Stressors**									
20	Braithwaite et al., (2015)	**N = 57**; 51 White, 4 Asian, 1 Black/African American, 1 Mixed	**2nd and 3rd trimester**; Range: N/A, Gestational Ages reported by subgroup, see column G	**Infant Distress Video** - a short film depicting distressed young infants, all under the age of 6 months. The film was 6 min in length and included 8 short clips of crying infants.	**5**	Slope calculated by subtracting the mean baseline cortisol measure from the 20-minute post-stimulus measure	**2 Groups: Control** (those scoring 9 and below on EPDS; Mean GA: 28.4 (SD=12.4)) and **Depressive-Symptom** (those scoring 10 or above on Edinburgh Postnatal Depression Scale; Mean GA: 29.9 (SD=6.67))	Mean change in salivary cortisol response to stressor was negative for both groups	**Mental Health Measure** - There were no group differences in prenatal salivary cortisol responses; **Trimester** - Days-of-gestation was not correlated with mean cortisol change in response to the stressor	No significant difference on measures of cortisol between women who did and did not report delivery complications
21	Braithwaite et al., (2016)	**N = 103**; 94 White, 7 Asian, 1 Black/African American, 1 Mixed, 1 Completed High School or Less	**2nd and 3rd trimester**; Range: 14.9–40.1 weeks, Mean GA: 27.2 (SD=NA)	**Infant Distress Video** - a short film depicting distressed young infants, all under the age of 6 months. The film was 6 min in length and included 8 short clips of crying infants.	**5**	Repeated measures ANOVAs	**2 Groups: Control** (those scoring 9 and below on EPDS) and **Depressive-Symptom** (those scoring 10 or above on Edinburgh Postnatal Depression Scale)	No change in salivary cortisol across the test session	**Mental Health Measure** & **Trimester** - There were no interactions between time, trimester and group, suggesting that participants from both groups and both the second and third trimester did not show a change in salivary cortisol across the test session	Significant between-subjects effect of trimester on salivary cortisol, with higher cortisol concentrations in the third trimester participants
22	Carbone et al., (2023)	**N = 39**; 12 White, 23 Black/African American, 4 Other, 10 Completed High School or Less	**2nd and 3rd trimeter**; Range: NA, Mean GA: 31.05 weeks (SD= 3.38)	**Baby Cry Protocol via Infant Simulator** - included the use of an infant simulator that was programmed using a pre-set frequent-cry schedule of human infant cry sounds.	**5**	Random Intercept and Random Slope Model - Process began with the estimation of an unconditional (i.e., intercept-only) model. Next, a random intercept was added to the estimation (i.e., random intercept model). This allows the baseline (i.e., intercept) cortisol value to differ across participants.	No groups within perinatal participants	Average cortisol values decreased from the first saliva sample to the fifth	No relevant variables	Cortisol levels for mothers with fewer Adverse Childhood Experiences (ACEs) were higher across the sample for those mothers who were in the later weeks of the third trimester of pregnancy. Among expectant mothers with 2 + ACEs, predicted baseline cortisol levels for expectant mothers later in the third trimester were the same or lower than levels for expectant mothers earlier in the third trimester. Mothers in the 0–1 ACEs groups, the average, predicted, baseline cortisol levels were higher across the sample throughout the third trimester, as is typical of pregnancy. Yet the predicted values for those in the 2 + ACEs groups appear to remain flat or were lower across the sample through the third trimester of pregnancy - suggesting decreased or blunted cortisol levels across pregnancy.
23	Derntl et al., (2015)	**N = 60**	**2nd and 3rd trimester**; Range: 16–37 weeks, Mean GA: 26.52 (SD=4.7)	**Fetal MRI**	**2**	Changes in cortisol levels were analyzed using an analysis of covariance for repeated measurements with the within-subject factor time (pre and post MRI) and the between-subject factor group (with or without accompanying person).	**2 Groups: Accompanied** and **Not Accompanied; 3 Groups: No Fetal Malformation, Fetal Pathology Compatible with Survival,** and **Fetal Pathology Probably Not Compatible with Survival**	Participants showed significant higher cortisol levels immediately before fetal MRI examination; all groups showed a decline in cortisol over time	No relevant variables	No significant correlation was obtained for skin conductance levels (SCL) and cortisol levels. There was no significant interaction between whether the participant was accompanied for the exam and cortisol levels. There was no significant effect observed between diagnosis severity and cortisol levels.
24	Evans et al., (2008)	**N = 182**; 19% White, 5% Asian, 10% Black/African American, 60% Hispanic/LatinX, 6% Mixed, 26% $0-$15,000, 17% $16,000-$25,000, 21% $26,000-$50,000,19% $51,000-$99,000, 12% $100,000-$250,000, 2% above $250,000	**3rd trimester**; Range: 33–39 weeks, Mean GA: 35.9 (SD= 1)	**Stroop task** - participants completed a Stroop color-word matching task and either mental arithmetic or controlled breathing task	**3**	Repeated measures ANOVA for baseline, anticipation (pre-task), reactivity (post sample)	**4 Groups: Control** (no current Axis I Diagnosis), **Depression** (current major depressive disorder episode or dysthymia), **Anxiety** (one or more current anxiety disorders), and **Comorbid** (currently met criteria for both a depressive and an anxiety disorder)	No significant within-subjects effect for time point on cortisol	**Mental Health Measure** - No significant interaction effect of time by diagnosis	Comorbid subjects had significantly higher salivary cortisol levels than controls but depressed and anxious participants did not differ significantly from controls. The second laboratory task (mental arithmetic versus paced breathing) did not differentially affect overall cortisol levels.
25	Fiterman and Raz, (2019)	**N** = **23**	**3rd trimester**; Range: 26–37 weeks, Mean GA: 32 (SD=3.69)	**Digit-symbol coding test, an arithmetic ability test, and a visual stop-signal task**	**2**	2 × 2 mixed-design ANOVA. Time (T1-base-line/T2–20 min post-test session) was the within-subject factor, and Group (pregnant/non-pregnant) was the between-subject factor	No groups within perinatal participants	Cortisol levels 20 min post-test session were significantly higher than baseline cortisol levels in both groups	No relevant variables	Pregnant women had lower cortisol levels than non-pregnant women. Cortisol reactivity was significantly more pronounced in non-pregnant women than in pregnant women. Cortisol reactivity was negatively correlated with Go trials, and positively correlated with error rates on Stop trials
26	Ghaemmaghami et al., (2014)	**N** = **34**	**2nd trimester**; Range: N/A, Mean GA: 15.96 weeks (SD= 0.71)	**Amniocentesis**	**6**	AUCi and ANOVA were used to assess possible interaction effects between condition and time. AUCi was computed with a slightly modified trapezoid formula to take morning decline in salivary parameters into account. Control variables were assessed before the main analyses based on bivariate correlations conducted between maternal characteristics and biological variables and included maternal BMI and gestational age	No groups within perinatal participants	The analysis of the AUCi revealed a significant increase in salivary cortisol during the amniocentesis	**Mental Health Measure** - No significant association was found between the maternal biological stress response and state anxiety (STAI-s); **Psychosocial Stress Measure** - No significant association was found between the maternal biological stress response and perceived stress (VAS)	No significant associations were found between the maternal psychological stress response and the maternal biological stress response (AUCi) of salivary cortisol (SalF). The SalF response to the stress of the amniocentesis (AUCi) was significantly and positively related to amniotic fluid E. Partial correlations revealed that the maternal SalF response was negatively associated with infant birth weight and size at birth.
27	Kammerer et al., (2002)	**N** = **10**	**3rd trimester**; Range: N/A, Mean GA: 36.8 weeks (SD=2.5)	**Cold-Hand Stressor Test** - the participants put their non dominant hand into ice cold water for one minute	**3**	Analysis by paired *t*-test comparing values at 0 and 20 min	No groups within perinatal participants	Pregnant women showed no significant mean increase in saliva cortisol 20 min after im_mersing the hand in ice cold water, despite a small increase in some individuals	No relevant variables	N/A
28	La Marca-Ghaemmaghami et al., (2013)	**N = 34**	**2nd trimester**; Range: 15–17.9 weeks, Mean GA: 15.9 (SD=0.7)	**Amniocentesis**	**6**	AUCi - a slightly modified trapezoid formula was calculated which included subtracting the skew AUC with respect to ground (AUCg) for the connection between the first and the last salivary measures	No groups within perinatal participants	N/A - did not specifically state whether there was a change in cortisol as a response to stressor	**Psychosocial Stress Measure** - Perceived emotional support, calmness, and wakefulness were uncorrelated with the salivary cortisol response to stress induced by the amniocentesis.	There was no significant differences between women with an increased risk for chromosomal abnormalities and women with no increased risk with regard to emotional support and the physiological stress response. Pregnant women, who had undergone an invasive medical procedure previously were in a significantly worse mood and exhibited decreased SalE (salivary cortisone) responses to the amniocentesis compared to women with no prior experience with invasive medical procedures.
29	La Marca-Ghaemmaghami et al., (2017)	**N = 34**; 14 Completed High School or Less	**2nd trimester**; Range: 15–17.9 weeks, Mean GA: 15.9 (SD=0.7)	**Amniocentesis**	**6**	AUCi - in order to control for the morning decline in salivary cortisol, a slightly modified trapezoid formula was calculated which included subtracting the skew AUC with respect to ground (AUCg) for the connection between the first and the last salivary measures	No groups within perinatal participants	N/A - did not specifically state whether there was a change in cortisol as a response to stressor	No relevant variables	There was no significant association between the acute maternal cortisol response (AUCi) and amniotic fluid corticotropin releasing hormone (CRH) or urocortin (UCN) levels
30	Laurent et al., (2018)	**N = 230**; 88% White, 99% non-Hispanic/LatinX	**2nd and 3rd trimester**; Range: N/A, Mean GA: N/A	**Stroop task** - participants completed a Stroop color-word test (5 min) after which they were given 5 min to recover, followed by a math task (5 min), and a final 5-min recovery period	**3**	Other Hierarchical linear modeling (HLM) - An initial model was run to obtain estimates (Empirical Bayes Coefficients) of each mother's HPA activation during pregnancy. Cortisol levels were modeled with an intercept, representing mean cortisol across the prenatal visits. Cortisol values were log-transformed to correct positive skew, and standardized residuals after controlling for collection time were used in analyses	No groups within perinatal participants	On average, there was no significant effect of the stroop test on cortisol levels in participants^1^	No relevant variables	Mothers with higher cortisol at 30 and 36 weeks gestation tended to show a more rapidly increasing postpartum symptom trajectory, resulting in higher depressive symptoms by the end of the first postnatal year
31	Murphy et al., (2015)	**N = 53**; 51 White, 2 Other	**1st and 2nd trimester**; Range: 11.7–17.6 weeks, Mean GA: 14.9 (SD=10)	**Infant Distress Video** - a short film depicting distressed young infants, all under the age of 6 months. The film was 6 min in length and included 8 short clips of crying infants.	**5**	Change from baseline cortisol scores were calculated for each group and time point by subtracting the average baseline cortisol level from the cortisol level at the three post-film time points. ANOVA and independent samples *t*-tests - A mean was taken of the two baseline cortisol measures, and a *t*-test was performed to check that this did not differ between the two groups.	**2 Groups: Control** (those scoring 9 and below on Edinburgh Postnatal Depression Scale) and **Depressive-Symptom** (those scoring 10 or above on Edinburgh Postnatal Depression Scale)	Change from baseline in salivary cortisol level for the depressed group was positive. The change from baseline in salivary cortisol for the control group was mostly negative	**Mental Health Measure** - Although both groups showed a similar increase in self reported state anxiety in response to the film, there was a significantly increased cortisol response in the depressed symptom group compared with the control group	Baseline cortisol concentration before the start of the film was not significantly different between the depressed symptom group and controls
32	Riddle et al., (2023)	**N = 49**; 41 White, 4 Asian, 1 Black/African American, 4 Hispanic/LatinX, 1 American Indian/NA, 2 Other; 1 $26,000-$50,000,17 $51,000-$100,000, 28 $101000-$250,000, 3 $250,000 +	**3rd trimester**; Range: N/A, Mean GA: 34 weeks (SD=1.1)	**Stroop task** - participants completed a traditional Stroop (disassociating word meaning from printed word color within time limitations) along with an exploratory pregnancy specific Stroop: this task uses words considered to be anxiety-provoking in pregnancy (e.g., miscarriage, blood, stillbirth) and colors. Stroop tasks were administered one after the other, and the order was randomized	**4**	To assess stress responsiveness, linear mixed effects models were used to assess whether a group time interaction affected variables of interest, including salivary cortisol	**2 Groups: Control** (those scoring 20 and below on the Perinatal Anxiety Screening Scale and/or no diagnosis of a current, active anxiety disorder) and **Anxiety** (a score ≥21 on the Perinatal Anxiety Screening Scale and/or a diagnosis of a current, active anxiety disorder by SCID or clinician using DSM-5 criteria)	Average cortisol levels did not change over time for both groups	**Mental Health Measure** - Cortisol did not differ between groups across time; **Psychosocial Stress Measures** - Cortisol in response to the stressor was not found to be associated with psychological measures and psychiatric diagnoses	Cortisol did not differ between groups across time. Neither cortisol measurements at baseline or in response to the stressor were found to be associated with heart rate variability changes or with psychological measures and psychiatric diagnoses
33	Turan et al., (2024)	**N = 44**; 37 Completed High School or Less; Income greater than expenses= 1, Income equal to expenses= 26, Income less than expenses= 17	**3rd trimester**; Range: N/A, Mean GA: 37 weeks (SD=N/A)	**Different Nonstress Test (NST) Device Noise Levels**	**3**	Separate 4 × 3 mixed-model ANOVAs were conducted for each stress parameter, with NST noise level (at four levels) and time (pretest, midtest, and posttest) as within-subject factors and groups (intervention vs control) as between-subjects factors	**4 Groups: Control** (volume off), **Intervention Group I** (1–35 db(a)), **Intervention Group II** (36–60 db(a)), and **Intervention Group III** (61 db (a) and above)	Cortisol levels increased across all groups during exposure to the sound (midtest)	No relevant variables	Cortisol levels increased with the sound level of the NST device and prolonged exposure to the sound

1 This study did not explicitly report whether cortisol responses to the stressor reflected an overall increase in cortisol levels. In this case, we inferred the presence or absence of cortisol reactivity based on model specifications and fit statistics. Specifically, when the addition of a linear slope representing change across samples within stress sessions did not improve model fit relative to an intercept-only model (e.g., *χ*^2^ test ns), we interpreted this as indicating no systematic within-session cortisol change attributable to the stressor.

## Appendix A

### Search Strings

#### PubMed

((“Stress, Physiological” [Mesh] OR “Stress, Psychological” [Mesh] OR “stressor*” [tiab] OR “stress” [tiab])

AND

(“Pregnant People” [Mesh] OR “Pregnancy” [Mesh] OR “Pregnancy Complications” [Mesh] OR “pregnanc*” [tiab] OR “pregnant” [tiab] OR “prenatal” [tiab] OR “antenatal” [tiab] OR “perinatal” [tiab])

AND

(“cortisol” [tiab] OR “Hydrocortisone” [Mesh] OR “hydrocortisone” [tiab] OR “hypothalamic pituitary adrenal” [tiab] OR “hypothalamic-pituitary-adrenal” [tiab] OR “HPA” [tiab] OR “Glucocorticoids” [Mesh] OR “glucocorticoid*” [tiab] OR “Glucocorticoids” [Pharmacological Action] OR “neuroendocrine” [tiab] OR “stress response” [tiab] OR “stress reactivity” [tiab]) AND (“Saliva” [Mesh] OR “saliva” [tiab] OR “salivary” [tiab]))

NOT (animals [mh] NOT humans [mh])

Publication Date: 1990-present

Language: English

Results: 558

Date Searched: 3/5/25

Retrieves 11/11 gold standard articles

#### Embase via Embase.com

(('physiological stress'/exp OR 'stressor*':ti,ab,kw OR 'stress':ti,ab, kw) AND ('pregnant person'/exp OR 'pregnancy'/exp OR 'pregnancy complication'/exp OR 'pregnanc*':ti,ab,kw OR 'pregnant':ti,ab,kw OR 'prenatal':ti,ab,kw OR 'antenatal':ti,ab,kw OR 'perinatal':ti,ab,kw) AND ('cortisol':ti,ab,kw OR 'hydrocortisone'/exp OR 'hydrocortisone':ti,ab,kw OR 'hypothalamic pituitary adrenal':ti,ab,kw OR 'hypothalamus hypophysis adrenal system'/exp OR 'hypothalamic-pituitary-adrenal':ti,ab, kw OR 'hpa':ti,ab,kw OR 'glucocorticoid'/exp OR 'glucocorticoid':ti,ab, kw OR 'neuroendocrine':ti,ab,kw OR 'stress response':ti,ab,kw OR 'stress reactivity':ti,ab,kw) AND ('saliva'/exp OR 'saliva':ti,ab,kw OR 'salivary': ti,ab,kw)

AND ('article'/it OR 'article in press'/it OR 'preprint'/it OR 'review'/ it))

NOT ('animal'/exp NOT 'human'/exp)

Publication Date: 1990-present

Language: English

Results: 614

Date Searched: 3/5/25

#### CINAHL Ultimate via EBSCO

((MH “Stress+”) OR (TI stressor* OR AB stressor*) OR (TI stress OR AB stress))

AND

((MH “Expectant Mothers”) OR (MH Pregnancy+) OR (MH “Pregnancy Complications+”) OR (TI pregnanc* OR AB pregnanc*) OR (TI pregnant OR AB pregnant) OR (TI prenatal OR AB prenatal) OR (TI antenatal OR AB antenatal) OR (TI perinatal OR AB perinatal))

AND

((TI cortisol OR AB cortisol) OR (MH “Hydrocortisone”) OR (MH “Hypothalamic Pituitary Adrenal Axis”) OR (TI hydrocortisone OR AB hydrocortisone) OR (TI “hypothalamic pituitary adrenal” OR AB “hypothalamic pituitary adrenal”) OR (TI hypothalamic-pituitary-adrenal OR AB hypothalamic-pituitary-adrenal) OR (TI HPA OR AB HPA) OR (DE “Glucocorticoids”) OR (TI glucocorticoid OR AB glucocorticoid) OR (TI neuroendocrine OR AB neuroendocrine) OR (TI “stress response” OR AB “stress response”) OR (TI “stress reactivity” OR AB “stress reactivity”)) AND ((MH Saliva) OR (TI saliva OR AB saliva) OR (TI salivary OR AB salivary))

Publication Date: 1990-present

Source Type: Scholarly Journals

Results: 186

Date Searched: 3/5/25

#### PsycINFO via EBSCO

((DE “Stress” OR DE “Physiological Stress” OR DE “Psychological Stress” OR DE “Stress Reactions”) OR (TI stressor* OR AB stressor*) OR (TI stress OR AB stress))

AND

((DE “Pregnancy” OR DE “Adolescent Pregnancy” OR DE “Pregnancy Outcomes” OR DE “Primipara”) OR (TI pregnanc* OR AB pregnanc*) OR (TI pregnant OR AB pregnant) OR (TI prenatal OR AB prenatal) OR (TI antenatal OR AB antenatal) OR (TI perinatal OR AB perinatal))

AND

((TI cortisol OR AB cortisol) OR (DE “Hydrocortisone” OR DE “Cortisol Awakening Response”) OR (TI hydrocortisone OR AB hydrocortisone) OR (DE “Hypothalamic Pituitary Adrenal Axis”) OR (TI “hypothalamic pituitary adrenal” OR AB “hypothalamic pituitary adrenal”) OR (TI hypothalamic-pituitary-adrenal OR AB hypothalamic-pituitary-adrenal) OR (TI HPA OR AB HPA) OR (DE Glucocorticoids) OR (TI glucocorticoid OR AB glucocorticoid) OR (TI neuroendocrine OR AB neuroendocrine) OR (TI “stress response” OR AB “stress response”) OR (TI “stress reactivity” OR AB “stress reactivity”)) AND ((DE Saliva) OR (TI saliva OR AB saliva) OR (TI salivary OR AB salivary))

Publication Date: 1990-present

Language: English

Source Type: Scholarly Journals

Results: 313

Date Searched: 3/5/25

#### Web of Science Core Collection

TS= ((“stressor*” OR “stress”) AND (“pregnanc*” OR “pregnant” OR “prenatal” OR “antenatal” OR “perinatal”) AND (“cortisol” OR “hydrocortisone” OR “hypothalamic pituitary adrenal” OR “hypothalamic-pituitary-adrenal” OR “HPA” OR “Glucocorticoids” OR “glucocorticoid” OR “neuroendocrine” OR “stress response” OR “stress reactivity”) AND (“saliva” OR “salivary”))

Publication Date: 1990-present

Language: English

Citation Topics Meso: Exclude: 3.51 Dairy & Animal Sciences or 3.35 Zoology & Animal Ecology or 3.232 Veterinary Sciences

Document Types: Article, Review Article, Early Access

Results: 795

Date Searched: 3/5/25

#### Scopus

(TITLE-ABS-KEY(stressor*) OR TITLE-ABS-KEY(stress))

AND

(TITLE-ABS-KEY(pregnanc*) OR TITLE-ABS-KEY(pregnant) OR TITLE-ABS-KEY(prenatal) OR TITLE-ABS-KEY(antenatal) OR TITLE-ABS-KEY(perinatal))

AND

(TITLE-ABS-KEY(cortisol) OR TITLE-ABS-KEY(hydrocortisone) OR TITLE-ABS-KEY(“hypothalamic pituitary adrenal”) OR TITLE-ABS-KEY (hypothalamic-pituitary-adrenal) OR TITLE-ABS-KEY(HPA) OR TITLE-ABS-KEY(glucocorticoid) OR TITLE-ABS-KEY(neuroendocrine) OR TITLE-ABS-KEY(“stress response”) OR TITLE-ABS-KEY(“stress reactivity”)) AND (TITLE-ABS-KEY(saliva) OR TITLE-ABS-KEY(salivary))

Publication Date: 1990-present

Language: English

Keywords: Exclude: Swine, Animals, Animal, Animal Experiment Document Type: Article, Review

Results: 592

Date Searched: 3/5/25

#### Cochrane CENTRAL

IDSearch

#1MeSH descriptor: [Stress, Physiological] explode all trees

#2MeSH descriptor: [Stress, Psychological] explode all trees

#3stressor* OR stress

#4#1 OR #2 OR #3

#5MeSH descriptor: [Pregnant People] explode all trees

#6MeSH descriptor: [Pregnancy] explode all trees

#7MeSH descriptor: [Pregnancy Complications] explode all trees

#8pregnanc* OR pregnant OR prenatal OR antenatal OR perinatal

#9#5 OR #6 OR #7 OR #8

#10MeSH descriptor: [Hydrocortisone] explode all trees

#11MeSH descriptor: [Glucocorticoids] explode all trees

#12”cortisol” OR “hydrocortisone” OR “hypothalamic pituitary adrenal” OR “hypothalamic-pituitary-adrenal” OR “HPA” OR “Glucocorticoids” OR “glucocorticoid” OR “neuroendocrine” OR “stress response” OR “stress reactivity”

#13#10 OR #11 OR #12

#14MeSH descriptor: [Saliva] explode all trees

#15saliva OR salivary

#16#14 OR #15

#17#13 AND #16

#18#4 AND #9 AND #17

Publication Date: 1990-present

Language: English

Results: 162

Date Searched: 3/5/25
